# Drivers of bat researchers’ intent to adopt field hygiene practices

**DOI:** 10.1111/cobi.70252

**Published:** 2026-03-21

**Authors:** Joanna L. Coleman, Ewan A. Macdonald, Abigail L. Rutrough, Tanja M. Straka, Todd D. Little, Zachary Stickley, Tigga Kingston

**Affiliations:** ^1^ Human Dimensions Working Group, Bat Specialist Group (BSG), Species Survival Commission (SSC) International Union for Conservation of Nature (IUCN) Gland Switzerland; ^2^ Department of Biology Queens College at the City University of New York Flushing New York USA; ^3^ Graduate Center City University of New York New York New York USA; ^4^ Worcester College University of Oxford Oxford UK; ^5^ Department of Biological Sciences Texas Tech University Lubbock Texas USA; ^6^ Institute of Biology Freie Universität Berlin Berlin Germany; ^7^ Department of Educational Psychology, Leadership, & Counseling Texas Tech University Lubbock Texas USA; ^8^ Yhat Enterprises, LLC. Lubbock Texas USA; ^9^ Department of Evolution, Ecology and Behavior University of Illinois Urbana Illinois USA

**Keywords:** health risk, One Health, pathogen transmission, wildlife fieldwork, zoonotic disease, enfermedad zoonótica, One Health, riesgo sanitario, trabajo de campo con fauna, transmisión de patógenos, 人畜共患病, 病原体传播, 野生动物野外工作, 健康风险, 同一健康

## Abstract

Infectious disease is a growing threat to wildlife, with zoonotic transmission most likely at the human–wildlife interface. One underappreciated activity at this interface is fieldwork with wild animals, but associated risks can be mitigated through field hygiene (FH) practices, such as using personal protective equipment and other appropriate behaviors. Following the dissemination of International Union for Conservation of Nature FH guidelines for bat researchers, we investigated factors that affect bat researchers’ intent to use FH practices under a theory of planned behavior (TPB) framework. Under the TPB, a person's intent to perform a behavior is influenced by their attitude toward, their subjective norms around, and their perceived behavioral control (PBC) about the behavior. We invited researchers who had recently conducted bat‐related fieldwork to complete a qualitative questionnaire, generating data that we used to build a quantitative survey, which we disseminated widely to bat researchers. We analyzed ∼1000 survey responses with structural equation modeling and assessed the role of career stage, research focus, and socioeconomic status of the research location on intent. Bat researchers’ intent to adopt FH practices was high overall. For those who do not focus on disease projects, the subjective norm was a strong driver of intent, with mentors the most influential norm referents; authoritative bodies that set regulations and peers were influential too. The only modeled barrier to intent was PBC—with beliefs that FH practices are impractical or uncomfortable contributing most to PBC. We concluded that senior researchers should be encouraged to use FH practices and encourage their mentees to do likewise. Technical solutions and education to mitigate impracticality and discomfort issues should also be encouraged. Although we focused on bat researchers, all wildlife fieldwork entails pathogen transmission risks. To mitigate them, FH practices must become entrenched in the wildlife research community; achieving this goal requires regulatory and social measures.

## Abstract (es)

Factores que impulsan la intención de los investigadores de murciélagos por adoptar prácticas de higiene en el campo

**Resumen:**

Las enfermedades infecciosas son una amenaza creciente para la fauna silvestre, lo que vuelve más probable la transmisión zoonótica en la interfaz entre los seres humanos y la fauna. Una actividad subestimada en esta interfaz es el trabajo de campo con animales silvestres, pero los riesgos asociados pueden mitigarse mediante prácticas de higiene de campo (HC), como el uso de equipos de protección personal y otros comportamientos adecuados. Tras la difusión de las directrices de la Unión Internacional para la Conservación de la Naturaleza sobre HC para investigadores de murciélagos, investigamos los factores que afectan a la intención de los investigadores de murciélagos de seguir las prácticas de HC en el marco de la teoría del comportamiento planificado (TCP). Según la TCP, la intención de una persona de realizar un comportamiento está influenciada por su actitud hacia él, sus normas subjetivas al respecto y su control conductual percibido (CCP) sobre el comportamiento. Invitamos a investigadores que habían realizado recientemente trabajo de campo relacionado con los murciélagos a completar un cuestionario cualitativo, lo que generó datos que utilizamos para elaborar una encuesta cuantitativa, que difundimos ampliamente entre los investigadores de murciélagos. Analizamos ∼1000 respuestas a la encuesta con modelos de ecuaciones estructurales y evaluamos el papel que desempeñaban en la intención la etapa profesional, el enfoque de la investigación y la situación socioeconómica del lugar de investigación. La intención de los investigadores de murciélagos de adoptar prácticas de HC era alta en general. Para aquellos que no se centran en proyectos relacionados con enfermedades, la norma subjetiva era un fuerte impulsor de la intención, siendo los mentores los referentes normativos más influyentes; los organismos autorizados que establecen las regulaciones y los compañeros también eran influyentes. La única barrera modelada para la intención fue la CCP, con la creencia de que las prácticas de HC son poco prácticas o incómodas, lo que contribuye en mayor medida a la CCP. Llegamos a la conclusión de que se debe animar a los investigadores sénior a utilizar prácticas de salud con los murciélagos y fomentar que sus discípulos hagan lo mismo. También se deben fomentar las soluciones técnicas y la educación para mitigar los problemas de impracticabilidad e incomodidad. Aunque nos centramos en los investigadores de murciélagos, todo el trabajo de campo con fauna conlleva riesgos de transmisión de patógenos. Para mitigarlos, las prácticas de salud de los murciélagos deben arraigarse en la comunidad de investigación de fauna; para lograr este objetivo se requieren medidas normativas y sociales.

## 蝙蝠研究者采用野外卫生措施意愿的驱动因素

**【摘要】** 传染病对野生动物构成了日益严重的威胁, 而人畜共患病传播最可能发生于人与野生动物接触的地方。在这种接触中, 一项被低估的活动是针对野生动物的野外研究工作, 但通过实施野外卫生措施(如使用个人防护装备及其他适当行为)可降低相关风险。在国际自然保护联盟发布蝙蝠研究者野外卫生指南后, 我们基于计划行为理论框架, 探究了影响蝙蝠研究者采用野外卫生措施意愿的因素。计划行为理论框架认为, 个体实施行为的意愿受其对行为的态度、相关主观规范, 以及自我行为控制感的影响。我们邀请了近期开展蝙蝠相关野外工作的研究者完成了一份定性调查问卷, 由此获得了构建定量调查问卷的数据, 并将该定量调查问卷广泛分发至蝙蝠研究者群体。研究通过结构方程模型分析约1000份调查结果, 评估了职业阶段、研究方向及研究地点社会经济状况对研究者意愿的影响。结果显示, 蝙蝠研究者采用野外卫生措施的意愿总体较高。对于不关注疾病项目的研究者而言, 主观规范是意愿的重要驱动因素, 其中导师是最具影响力的规范参照者;制定规章的权威机构和同行也具有显著影响力。模型显示唯一阻碍意愿的因素是自我行为控制感, 认为野外卫生措施不切实际或不舒适是影响其自我行为控制感的主要因素。我们建议鼓励资深研究者采用野外卫生措施, 并引导其指导对象遵循相同做法。同时还应推广技术解决方案和教育培训以缓解可操作性与舒适度问题。尽管本研究聚焦蝙蝠研究者, 但所有野生动物野外工作均存在病原体传播风险。为降低相关风险, 必须在野生动物研究学术群体中形成野外卫生措施的共识, 实现这一目标还依赖于监管与社会措施。**【翻译:胡怡思;审校:聂永刚】**

## INTRODUCTION

Because the One Health concept recognizes that the health of humans, wildlife, and ecosystems is inextricably linked, wildlife conservation is integral to One Health (Zinsstag et al., 2011). Meanwhile, conservation biology often involves One Health risks due to its strong reliance on biological fieldwork (Soulé, 1985) (i.e., researchers working directly with living organisms *in situ*) (Ramírez‐Castañeda et al., 2022). Although remote methods (e.g., passive monitoring) and noninvasive surveys are vital to conservation biology (Lahoz‐Monfort & Magrath, 2021; Zwerts et al., 2021), fieldwork often involves capturing and handling wild animals, increasingly so with the rising use of physiological approaches to conservation (Lennox & Cooke, 2014; Madliger et al., 2021). Thus, wildlife fieldwork epitomizes the human–wildlife interface—typified by direct contact or close proximity between humans and nonhuman animals—that is central to the One Health paradigm (Dreyer et al., 2023).

A core One Health concern related to wildlife fieldwork is pathogen transmission between species (Gebreyes et al., 2014). After all, most emerging infectious diseases that affect humans originated in wild animal populations—increasingly so since the 1940s (Jones et al., 2008)—with biodiversity loss and climate change exacerbating the risk of disease spillover (Pfenning‐Butterworth et al., 2024). Biodiversity loss is also increasingly driven by emerging infectious diseases, such as canine distemper, sarcoptic mange, chytridiomycosis (Daszak et al., 2000; Fisher et al., 2020; Smith et al., 2009), and avian influenza (Lambertucci et al., 2025). Given the core mission of conservation biology—preventing species extinctions (Soulé, 1985)—conservation researchers who do fieldwork with wildlife should exhibit a fundamental concern with limiting negative impacts of their work.

The One Health risks of wildlife fieldwork entail three disease transmission pathways. The first is wildlife‐to‐human transmission: researchers become infected by a zoonotic pathogen. This has happened. Some cases—such as histoplasmosis (Cottle et al., 2013)—resolved, whereas others—such as hantavirus (Sinclair et al., 2007), pneumonic plague (Wong et al., 2009), and rabies (Jakava‐Viljanen et al., 2010)—had fatal outcomes. So far, there are no records of pathogen spillovers during wildlife fieldwork precipitating person‐to‐person transmission. But as the COVID‐19 pandemic (Crits‐Christoph et al., 2024) and H5N1 influenza outbreak (Peacock et al., 2025) have shown, the evolution of zoonotic pathogens in human hosts can have dramatic public health effects.

The second risk is human‐to‐wildlife transmission: researchers transmit a pathogen directly to a wild animal or introduce a pathogen into a habitat where it infects wild hosts. This scenario is also called pathogen pollution (Cunningham et al., 2003), and it has increased in recent decades (Alexander et al., 2018; Goldberg et al., 2024), notably in the case of chytridiomycosis, which has caused the extinction of many amphibian species (Fisher et al., 2020). This risk has also played out during wildlife fieldwork, especially involving nonhuman primates, with many putative or confirmed cases (including some fatal) of great apes contracting human‐borne pathogens as a result of contact with people, including researchers (Dunay et al., 2018; Fagre et al., 2022; Messenger et al., 2014).

The third risk is wildlife‐to‐wildlife transmission: researchers facilitate pathogen transmission between individual wild animals by holding them together for short or prolonged periods of captivity or by causing them to aggregate unnaturally or touch the same fomites. For example, wildlife baits and lures, which conservation researchers often use to attract and sample target animals (Dehaudt et al., 2024), raise the risk of pathogen transmission (Sorensen et al., 2014). And research has shown that researchers can spread ranaviruses among amphibians if they do not take adequate biosecurity measures (Gray et al., 2018).

Recognizing the elevated risks of multidirectional pathogen transmission during wildlife fieldwork, various regulatory agencies have set mitigative guidelines. Some guidelines target specific pathogens, such as biosafety practices that aim to prevent the spread of chytrid fungus in amphibians (Canadian Herpetofauna Health Working Group, 2017; Woodhams et al., 2021) or avian influenza in birds (U.S. National Science Foundation, 2023). Other guidelines address specific settings. For example, the U.S. National Parks Service developed its “Safe Work Practices for Employees Handling Wildlife” after the above‐cited case of pneumonic plague (Bosch et al., 2013). Yet, there remains little to no standardization of biosecurity guidelines for wildlife fieldwork (and see Woodhams et al., 2021) and, in many cases, no guidelines or oversight.

We addressed the One Health risks linked to fieldwork with bats (order Chiroptera), an endeavor that illustrates all three transmission pathways above and often involves directly capturing and temporarily holding large numbers of individuals. For example, three of us (J.L.C., T.M.S., T.K.) have routinely captured dozens of bats at once in a single net or harp trap and, prior to the release and dissemination of the IUCN FH guidelines, placed 10 or more individuals in one holding bag to await processing. Many bat researchers also use acoustic lures to increase capture success (e.g., Braun De Torrez et al., 2017; Hill & Greenaway, 2005; Preble et al., 2021). The risk of bat‐to‐human pathogen transmission may be heightened by the fact that bats, as a taxon, can host very diverse microbes. However, bats do not host proportionately more numerous or deadlier pathogens than other taxa (Mollentze & Streicker, 2020), disease prevalence is low in many bat populations (e.g., Loh et al., 2022), and most bat‐borne microbes are ill‐adapted to infecting humans (Van Brussel & Holmes, 2022). Still, some bat species are the natural reservoirs of virulent zoonoses (e.g., Nipah, Hendra, Marburg) or the putative ancestral hosts of others (Crits‐Christoph et al., 2024), and bat researchers have occasionally been infected by bat‐borne pathogens (e.g., *Sosuga virus* [Amman et al., 2015]) during fieldwork. Thus, bat‐to‐human pathogen transmission during fieldwork, though relatively rare, could have severe outcomes.

The risk of bat‐to‐bat pathogen transmission is generally lessened by the precautions that bat researchers take (such as collecting tissues with sterile instruments) to prevent sample cross‐contamination (Aguilar‐Setién et al., 2022). But bat researchers often also put individual bats in contact with the same fomites (e.g., nets, tools, processing surfaces) or in common holding bags, promoting both intra‐ and interspecific pathogen transmission.

The risk of pathogen pollution is illustrated by white nose syndrome (WNS). This disease, which affects hibernating bats and is causing the worst wildlife epidemic in North American history, is caused by the fungus *Pseudogymnoascus destructans* (Cheng et al., 2021), which was introduced to North American caves by people (Leopardi et al., 2015). A recent concern—that people might transmit SARS‐CoV‐2 to naïve bat populations (e.g., Gryseels et al., 2021; Olival et al., 2020)—seems to have manifested in the United States, where eastern red bats (*Lasiurus borealis*) were among six mammal species that tested positive for SARS‐CoV‐2 (Goldberg et al., 2024). Here, the risk is not only that infected bats could become ill but also that affected bat populations could become novel viral reservoirs, leading to an elevated public health risk (Fagre et al., 2022; Olival et al., 2020). One possible consequence of such a scenario is (unwarranted) retaliatory persecution of bats, as observed early in the COVID‐19 pandemic (Nanni et al., 2022). Persecution of bats (stemming from diverse human–bat conflicts) is already a major conservation threat to bats globally (Frick et al., 2020); any further implication of bats in public health risks would compound it.

In this context, the Bat Specialist Group (BSG) of the International Union for Conservation of Nature (IUCN) Species Survival Commission (SSC) developed and published mitigative guidelines for bat researchers (available at https://www.iucnbsg.org/bsg‐publications.html). Version 1.0 (2020) greatly reduced all bat research and introduced so‐called basic field hygiene (FH) practices that minimize multidirectional (human‐to‐bat, bat‐to‐human, bat‐to‐bat) pathogen transmission by reducing exposure to bat body fluids and aerosols. Version 2.0 (2021) invited a greater return to fieldwork via risk assessment and adoption of FH practices. Both versions were superseded by Shapiro et al. (2024), which was disseminated throughout the bat research community and adopted by agencies governing bat research worldwide. It identifies FH practices that we grouped into four categories: sanitary processing (regularly disinfecting surfaces, measuring equipment, cleaning field gear between locations); good behaviors (not eating, drinking, or smoking near bats, using an air‐puffing tool instead of blowing on bats, which is a common practice to part fur and see the skin or stop bats from biting); personal protective equipment (PPE) (using masks, gloves, and dedicated field clothes); and vaccination (having updated prophylactic rabies and COVID‐19 vaccines).

The FH practices, despite being quite simple, represent a major departure from how many, if not most, bat researchers (except those who study diseases) were trained and have worked (J.L.C., T.M.S., T.K., personal observation; Appendix S1). Compounding the human tendency to resist change (Oreg, 2003) is the perception that many FH practices are awkward and uncomfortable and increase handling time and cost. Further, the low transmission risk may be perceived as negligible, especially by researchers who, after decades of experience with no known incidents, become cavalier. Hence, some resistance by bat researchers and the need for interventions (e.g., outreach, training) to increase adherence to the guidelines are expected. Moreover, with fieldwork generally done at times and places that preclude direct regulatory oversight, understanding how to motivate bat researchers to use FH practices is essential.

**FIGURE 1:**
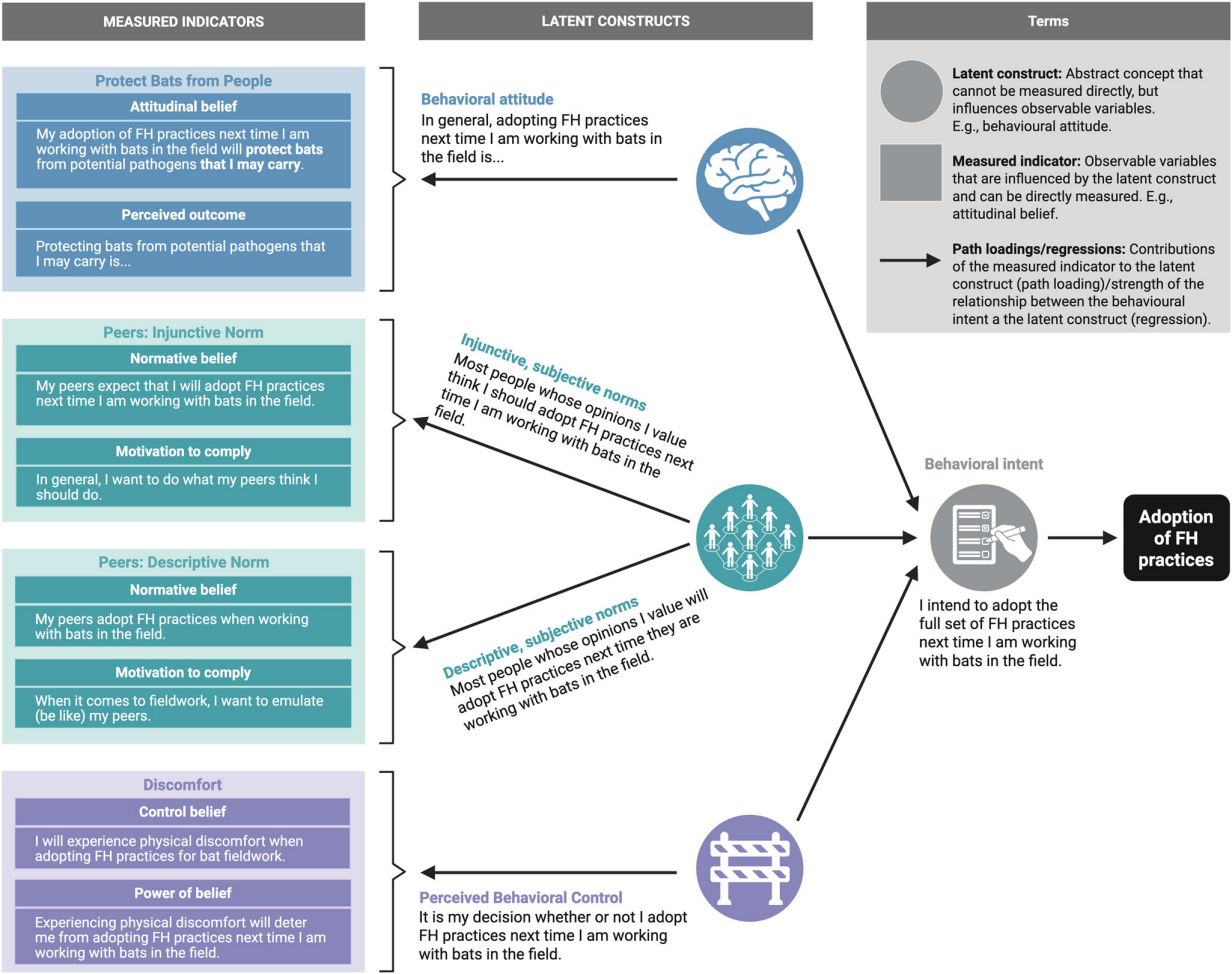
Use of the theory of planned behavior approach in a study of bat researchers’ intent to adopt field hygiene (FH) behaviors. Survey items were presented in couplets, each consisting of a belief statement paired with a statement measuring the perceived impact of the belief on the intent to perform the focal behavior. A subset of couplets is illustrated. For example, our survey included four behavioral attitude couplets assessing the motivations to protect: people from bats, bats from people, and bats from bats; and to minimize cross‐contamination.

To build this understanding and inform future interventions, we investigated bat researchers’ intent to adopt FH practices. Our conceptual framework was Ajzen's (2020) theory of planned behavior (TPB), which is widely used to study the drivers of and barriers to behaviors, including those related to conservation (e.g., Kaiser et al., 2005) and human health (e.g., Bosnjak et al., 2020). The TPB posits that whether someone performs a focal behavior is directly predicted by their intent to do so (behavioral intent) (hereafter, *intent*), which has three direct antecedents (Ajzen, 2020). First, their behavioral attitude (hereafter, *attitude*) reflects their beliefs about the value and outcome of the behavior. Second, their subjective norms (hereafter, *norm*) reflect their beliefs about whether the entities—referents—whose opinions they value expect them to practice the behavior (injunctive norm) and practice it themselves (descriptive norm); the influence on intent is modified by the person's motivation to comply with said norms. Third, their perceived behavioral control (PBC) reflects how easy or difficult they believe it is to perform the behavior and the power of PBC to affect their intent (Figure 1).

We tested the applicability of the TPB framework to the adoption of FH practices both generally and in reference to more specific categories (sanitary processsing, good behaviors, wearable PPE, vaccines) and evaluated whether and how the relative contribution of these antecedents varies with researchers’ demographics and the type of research they do. We tested three hypotheses. First, junior researchers are the most responsive to norms because the seeking of external validation and the desire to belong decline with age and seniority (H1) (Sinclair & Agerström, 2023). Second, the intent to adopt FH practices is highest among researchers who do disease‐related research because such practices are more ingrained in their culture, training, and authoritative guidelines (H2). Should this be true, we were particularly interested in explaining variation in intent among nondisease researchers (the likeliest targets of future interventions). Third, the influence of PBC rises in low‐income countries, where PPE items may be less affordable and accessible and, because most of these countries are in the tropics, heat and humidity make wearing PPE more uncomfortable (H3).

Finally, we hypothesized that intent varies among the four categories of FH practices (sanitary processing, good behaviors, wearable PPE, vaccines) (H4). For example, the intent to be vaccinated might be almost universal considering the health risks of not doing so and mandatory vaccination programs. In contrast, the intent to use PPE might be much more variable. We refer to these as specific FH intents, contrasting with the general intent to adopt all FH practices.

## METHODS

We used an exploratory, sequential mixed‐methods study design. To develop a contextually relevant quantitative survey, we first deployed a qualitative survey to establish which of the recommended FH practices bat researchers used and to generate a set of statements about their common attitudes, norms, and PBCs.

### Qualitative survey

To identify researchers who had recently led fieldwork involving close proximity (<2 m) to live, wild bats, we searched Web of Science (WoS) for journal articles published in 2020 and 2021 with *Chiroptera* in the topics (*n* = 539). To retain studies that clearly involved contact with live bats, we scanned the titles, abstracts, and methods sections, generating a pool of 290 records. We reduced geographic bias by using WoS country‐level analytics to stratify our pool and chose the first three records per represented nation. We emailed each paper's corresponding author and asked this person to complete the survey if they personally did the fieldwork or, if not, to forward our request to the person who had done so.

We used Qualtrics to disseminate our online survey, whose items mapped to the TPB (Appendix S2). For example, Q2.2 asked which FH practices they adopt to keep themselves and bats safe (intent) and how important they believe them to be (attitude); Q2.5 and 2.7 sought to identify referent groups (norms), with Q2.7 also asking what makes it difficult for respondents to adopt FH practices (PBC). We collected 84 responses, from 13 September through 1 November 2021, a period that shortly followed the dissemination of Version 2.0 of the BSG guidelines (8 July 2021). Thus, our respondents could have had two exposures to the guidelines, both versions of which were rolled out via social media campaigns and emails to all BSG members.

### Quantitative survey

After coding all qualitative data into unique bins and generating a frequency distribution for each question, we used the most common bins (FH_practices_data_item1) to build our quantitative survey (Appendix S3), which we distributed through Qualtrics. For example, the top four beliefs about the value of FH practices (Q2.2 in the qualitative survey) were (in descending order) that they: protect people, protect bats, minimize bat‐to‐bat disease transmission, and minimize sample contamination. Or, when asked what makes it difficult for respondents to adopt FH practices (Q2.7), the top three responses were: discomfort, costs, and hindrance of tasks.

Our instructions to respondents included a definition of fieldwork and a summary of the BSG guidelines with a hyperlink to the full guidelines (Version 2.0). We built 7‐point Likert scale items and presented them in couplets (following Ajzen, 2019) (Figure 1). We paired each attitude belief item with an item measuring the strength of that belief (e.g., Q8/Q9), each norm belief item with an item measuring motivation to comply (e.g., Q17/Q18), and each PBC belief item with an item measuring the power of that belief (e.g., Q27/Q28). We measured participants’ intent to adopt any of 13 specific FH practices (Q35), which we used to build four focused constructs corresponding to the four specific FH practice categories (sanitary processing, good behaviors, wearable PPE, vaccines), and their demographics to test hypotheses H1 through H3.

Before deploying our survey, we conducted cognitive interviews with six people from different nations (Canada, Germany, India, Taiwan, Philippines, Nigeria) and with variable English‐language skills to ensure that our items were widely understandable. We performed a pilot study (*n* = 45 respondents) in August 2022 and revised problematic items before launching our full survey.

### Data collection

Our survey ran from 13 September 2022 through 7 February 2023. Recruitment was purposive, with invitations disseminated through social media accounts and email lists of organizations whose audiences were highly likely to include individuals conducting bat fieldwork. Specifically, we advertised the survey on the Twitter and Facebook accounts of the Global Union of Bat Diversity Networks (GBatNET). We also emailed invitations to the >600 attendees of the 2022 joint International Bat Research Conference and annual meeting of the North American Society for Bat Research, to all BSG members, and to leaders of GBatNET's member networks. This approach maximized our ability to contact a geographically diverse sample of bat researchers. Recruitment involved self‐selection of participants and snowball‐like dissemination, as recipients were invited to forward the survey to other relevant individuals. Finally, we emailed single reminders to the original BSG and GBatNET recipients and to researchers in underrepresented regions (e.g., East Asia) to encourage broader geographic diversity in the sample. We set Qualtrics parameters to ensure a fully anonymized dataset.

Each participant provided informed consent, and this study was approved by the Institutional Review Board of Texas Tech University under IRB2021‐454 (qualitative study) and IRB2022‐389 (quantitative survey).

### Data cleaning

In all, 999 participants took our survey (partial responses included). We combined and reclassified data into groups used in later analyses. We classified respondents’ fieldwork locations into IUCN regions (https://iucn.org/our‐union/commissions/world‐commission‐protected‐areas/about/regions) and categorized each region's socioeconomic status (SES) as high or low based on the World Bank 2023 classifications of countries (https://datacatalogapi.worldbank.org/ddhxext/ResourceDownload?resource_unique_id=DR0090754&_gl=1*1fmc442*_gcl_au*MTE1NTA1OTQwNC4xNzI0MTcwMzI4).

### Analyses

We used structural equation modeling (SEM) to analyze our data. The SEM approach models the ability of latent constructs (theoretical hypotheses that cannot be directly measured but can be modeled using multiple measured indicators) to predict an outcome by blending regression and confirmatory factor analysis (CFA) (Figure 1). In summary, we first built a core model that served as the scaffold for all additional models. This core model was functionally two models, with one‐half regressed onto the other to create a single model. We built one‐half with the TPB questionnaire and corresponding latent constructs. For the other half, we used specific FH constructs, which we created by combining highly covarying FH indicators (responses to Q35) in an intuitive way to create post hoc constructs of FH behaviors. Finally, we built multigroup models to test how these relationships were affected by respondents’ career stage (H1), research focus (H2), and SES of their fieldwork location (H3).

We determined the mean for each couplet from our survey (attitudinal belief and outcome, normative belief and motivation to comply, control belief and power of control) (following Little et al. [2013]). All couplet pairs were positively correlated (*p* < 0.01) (Appendix S4). To avoid issues of collinearity between the PBC and norm constructs that were present in initial modeling efforts, we regressed the indicators of norm onto the indicators of PBC and saved the residuals, which we then used as residualized indicators of PBC. These residualized indicators represent all the variance of PBC that is independent of norm (Little et al., 2006).

We ran an initial CFA on the full dataset to fit an initial measurement model in three steps. First, we built the TPB's latent constructs (attitude, norm, PBC) from the measured indicators (Figure 1). Second, we created specific FH constructs by combining highly covarying FH indicators. Third, we regressed the FH constructs onto the TPB constructs to create the full structural model that evaluated predictors of general and specific intents. We used effects coding to fit this and all subsequent multigroup models so that the intercepts of the latent constructs would be on the same 7‐point Likert scale as that of the survey items (following Little et al. [2006]).

After running this initial single‐group model on the full dataset (hereafter, *full model*), we ran three multigroup models to test for significant intergroup differences that we had predicted in relation to career stage (student, early career, mid‐career, late career), research type (disease, nondisease, both), and SES of fieldwork location (high, low, both). Before testing for these differences, we tested measurement invariance to ensure that the items used to measure each latent construct performed equivalently in each group. After establishing invariance or partial invariance, we tested for intergroup differences, pruned nonsignificant regressions, and tested whether significant regressions were different between groups with nested chi‐square tests of significant reduction in model fit at each step.

Depending on the model, there were 40–42 missing data points and 30–35 missing data patterns (i.e., configurations in which, e.g., two rows with the same configuration of missing data are considered one data pattern). We used full information maximum likelihood to estimate the model based on the information present in other observations, thereby adjusting our parameter estimates for any potential bias caused by missing observations and retaining the power of our full sample size (Enders, 2022; Little et al., 2013). We fitted all models with the Lavaan package 0.6‐16 (Rosseel, 2012) in R 4.2.1 (R Core Team, 2022).

## RESULTS

Our respondents had a mean age of 41 years, 51.7% self‐identified as male, and early‐career researchers were the largest group (31.4%). The dominant regions in our sample were North America (where 31.5% of respondents resided; named as the primary fieldwork location 162 times) and western Europe (where 20.4% of respondents resided; named as the primary fieldwork location 103 times). Raw general intent to adopt FH practices was high, with a mean score of 5.72 (scale of 1–7) (details in Appendices S5 & S6).

The full model (*n* = 667) had excellent predictive power and fit (comparative fit index = 0.932, Tucker–Lewis index = 0.919, root mean square error of approximation = 0.049, standardized root mean squared residual = 0.045), capturing 41.7% of variation in general intent (*R* ^2^ = 0.417). The most influential predictors were the subjective norm, which was positively associated with general and specific intents, and PBC, which negatively influenced general and specific intents (Table 1). Attitude was less influential and only predicted general intent (Table 1). As hypothesized, intent varied among categories of FH practices.

**TABLE 1:** Regression results from full and multigroup structural equation models testing the influence of attitude, norms, and perceived behavioral controls on five field hygiene behavioral outcomes.

	Attitude			Norm			Control			
	Estimate	Standardized	*p*	Estimate	Standardized	*p*	Estimate	Standardized	*p*	*R* ^2^
Full model										
General intent	0.326	0.178	0.002	0.515	0.382	<0.001	−0.613	−0.347	<0.001	0.417
Sanitary processing	–	–	–	0.445	0.376	<0.001	−0.343	−0.221	<0.001	0.191
Good behaviors	–	–	–	0.404	0.404	<0.001	−0.670	−0.513	<0.001	0.426
PPE	–	–	–	0.576	0.610	<0.001	−0.356	−0.289	<0.001	0.456
Vaccines	–	–	–	0.153	0.150	0.004	−0.208	−0.156	0.002	0.047
Career stage										
Student										
General intent	–	–	–	0.567^2^	0.455	<0.001	−0.485^3^	−0.388	<0.001	0.358
Sanitary processing	–	–	–	0.427^4^	0.393	<0.001	–	–	–	0.154
Good behaviors	–	–	–	0.589^6^	0.507	<0.001	–	–	–	0.257
PPE	–	–	–	0.927^8^	0.680	<0.001	–	–	–	0.462
Vaccines	–	–	–	–	–	–	–	–	–	–
Early career										
General intent	0.299^1^	0.216	0.001	0.567^2^	0.410	<0.001	−0.485^3^	−0.350	<0.001	0.479
Sanitary processing	–	–	–	0.427^4^	0.384	<0.001	−0.230^5^	−0.207	<0.001	0.190
Good behaviors	–	–	–	0.589^6^	0.450	<0.001	−0.603^7^	−0.461	<0.001	0.415
PPE	–	–	–	0.927^8^	0.653	<0.001	−0.392^9^	−0.277	<0.001	0.503
Vaccines	–	–	–	–	–	–	–	–	–	–
Mid−career										
General intent	0.299^1^	0.215	0.001	0.567^2^	0.408	<0.001	−0.485^3^	−0.349	<0.001	0.482
Sanitary processing	–	–	–	0.427^4^	0.384	<0.001	−0.230^5^	−0.207	<0.001	0.190
Good behaviors	–	–	–	0.589^6^	0.450	<0.001	−0.603^7^	−0.461	<0.001	0.415
PPE	–	–	–	0.927^8^	0.653	<0.001	−0.392^9^	−0.277	<0.001	0.503
Vaccines	–	–	–	0.318^10^	0.291	<0.001	−0.298^11^	−0.273	<0.001	0.160
Late career										
General intent	–	–	–	0.567^2^	0.455	<0.001	−0.485^3^	−0.388	<0.001	0.358
Sanitary processing	0.689	0.567	<0.001	–	–	–	–	–	–	0.322
Good behaviors	–	–	–	0.589^6^	0.450	<0.001	−0.603^7^	−0.461	<0.001	0.415
PPE	0.545	0.479	0.003	–	–	–	–	–	–	0.229
Vaccines	–	–	–	0.318^10^	0.291	<0.001	−0.298^11^	−0.273	<0.001	0.160
Research type										
Not disease related										
General intent	–	–	–	0.593^2^	0.473	<0.001	−0.470^3^	−0.375	<0.001	0.364
Sanitary processing	–	–	–	0.438^4^	0.392	<0.001	−0.236	−0.211	<0.001	0.199
Good behaviors	–	–	–	0.529	0.401	<0.001	−0.678	−0.514	<0.001	0.425
PPE	–	–	–	0.876^5^	0.639	<0.001	−0.337	−0.246	<0.001	0.469
Vaccines	0.174	0.171	0.01	–	–	–	–	–	–	0.029
Disease related										
General intent	0.450^1^	0.410	<0.001	–	–	–	–	–	–	0.168
Sanitary processing	–	–	–	–	–	–	–	–	–	–
Good behaviors	–	–	–	–	–	–	–	–	–	–
PPE	–	–	–	–	–	–	–	–	–	–
Vaccines	–	–	–	–	–	–	–	–	–	–
Both										
General intent	0.450^1^	0.297	<0.001	0.593^2^	0.392	<0.001	−0.470^3^	−0.311	<0.001	0.563
Sanitary processing	–	–	–	0.438^4^	0.401	<0.001	–	–	–	0.161
Good behaviors	1.77	0.870	0.569^a^	–	–	–	–	–	–	0.758
PPE	–	–	–	0.876^5^	0.659	<0.001	–	–	–	0.434
Vaccines	–	–	–	–	–	–	–	–	–	–

*Note*: Shared superscripted numbers indicate the regression is fixed between groups.

Abbreviation: PPE, personal protective equipment.

^a^ Pruning leads to significantly worse model fit.

We found significant intergroup differences for career stage (*p* = 0.009) and research type (*p* = 0.005) but not SES of research location (*p* = 0.196) (effect sizes, approximated with *R* ^2^‐values, in Table 1 [details in Appendix S7 and S8]). The norm positively influenced researchers’ general intent and most specific intents regardless of their career stage (Table 1). In line with H1 (junior researchers are most responsive to norms), students’ intents were almost fully predicted by the norm, whereas the intents of early‐, mid‐, and late‐career researchers were mainly influenced by both the norm and PBC. Attitude had little to no influence on the intent of students or early‐ and mid‐career researchers but was the only driver of late‐career researchers’ specific intents to use PPE and sanitary processing (Table 1).

**FIGURE 2:**
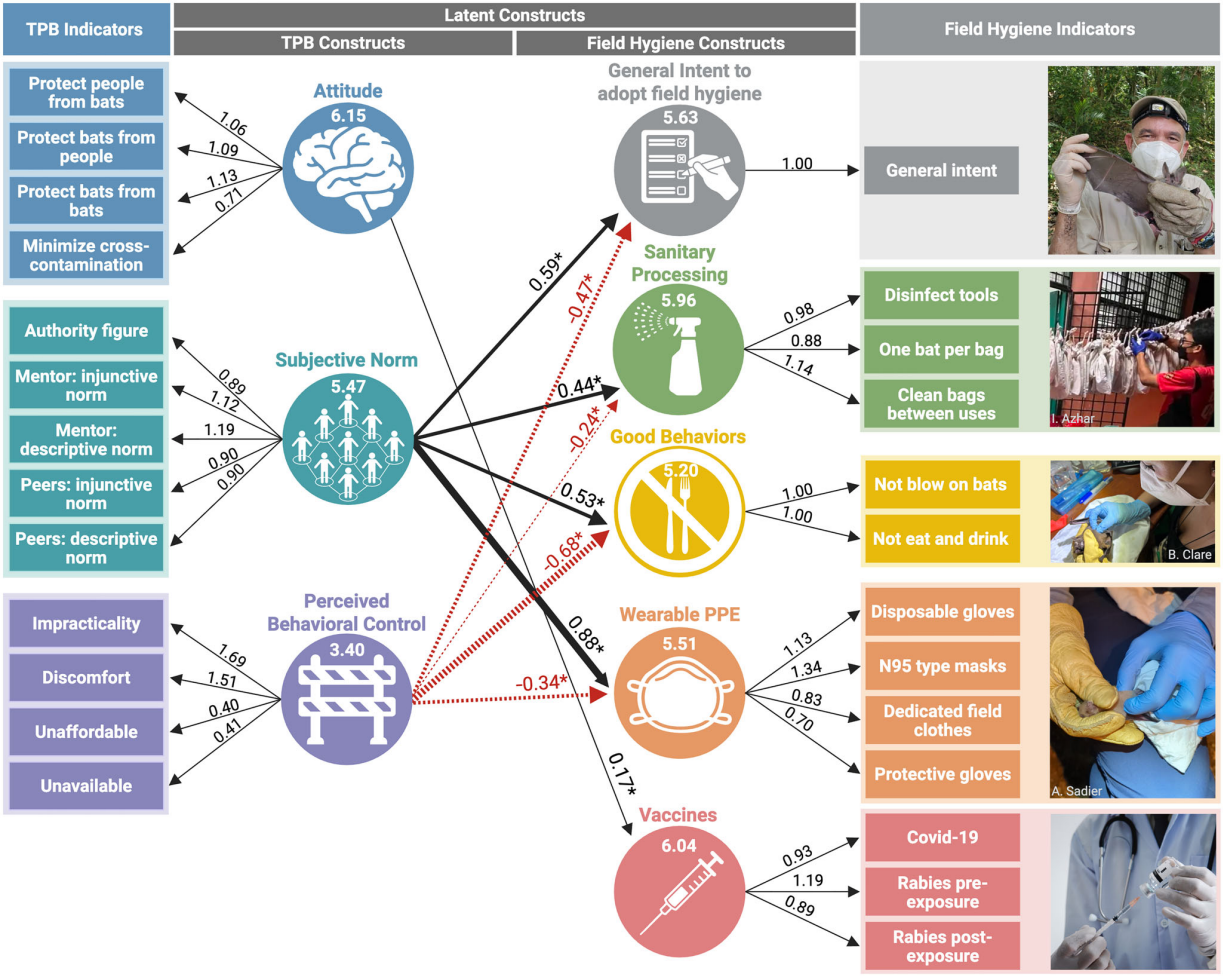
A structural equation model of drivers of the intent to adopt field hygiene (FH) behaviors by nondisease bat researchers (i.e., those who do not study disease questions), within the research‐type (disease vs. nondisease) multigroup model. Rectangles are the measured indicators used to describe the latent constructs in the center of the figure, latent constructs on the left are antecedents of behavioral intentions, latent constructs on the right are the specific FH intents; arrows from antecedents to behavioral intentions are significant regressions (p ≤ 0.01); solid black arrows, positive directionality; red dashed arrows, negative directionality; arrow widths and values above, correlation weights. For example, intent to use personal protective equipment was most influenced by a positive relationship with the subjective norm followed by a negative relationship with perceived behavioral control. Numbers in circles are latent mean responses on a seven‐point Likert scale.

In line with H2 (researchers who do disease‐related research have the highest intent to adopt FH practices), modeled intents to adopt general and specific FH practices were higher among disease researchers (general intent: 6.507 vs. 5.633; sanitary processing: 6.551 vs. 5.963; good behaviors: 5.867 vs. 5.199; PPE: 6.319 vs. 5.509; vaccines: 6.290 vs. 6.040 [details in Appendix S9]) and entirely driven by their attitudes (Table 1). For nondisease researchers, the norm and PBC were the main predictors; attitude only influenced their vaccine‐related intent (Table 1; Figure 2).

For SES of fieldwork location, geography mattered, but not how we expected. There was no difference between SES groups, and, contrary to H3 (affordability and access to PPE are greater barriers in low‐SES regions), the contributions of cost and access to PBC did not differ between high‐ and low‐income regions. Rather, beliefs that impracticality and discomfort are barriers to fieldwork were higher among researchers who only work in high‐SES locations (1.191 vs. −1.922; 0.908 vs. −1.432) (details in Appendix S10). This also contradicted our expectation that impracticality and discomfort are bigger barriers in low‐SES regions, which also tend to be hotter.

Zeroing in on nondisease researchers—the main target audience of, but least compliant with, FH guidelines—our model (Figure 2) captured 36.4% of variation in nondisease researchers’ general intent (*R* ^2^ = 0.364). The norm and PBC influenced the intent to adopt all FH practices except vaccines; only attitude predicted vaccine‐related intent. Generally, the norm was more influential than PBC—substantially so for sanitary processing and PPE—but PBC had more influence on their intent to adopt good behaviors (regression estimates in Table 1; Figure 2). The belief that FH practices minimize cross‐contamination contributed less to attitudes than did beliefs that they protect people from bats, bats from people, and bats from bats, whose contributions were functionally equal (path loadings; Figure 2). As to the most important norm referents, mentors mattered slightly more than peers or authority figures, who had equal contributions (Figure 2). However, the raw data suggest that researchers’ desire to comply with an authoritative body exceeds the belief that the authoritative body expects them to adopt FH practices (Appendix S4). Furthermore, the injunctive and descriptive norms set by mentors contributed functionally equally to mentees’ intent. The main contributors to PBC were beliefs that FH practices are impractical and uncomfortable (Figure 2).

## DISCUSSION

Overall, bat researchers expressed strong intentions to adopt FH practices, indicating that the One Health risks of bat‐related fieldwork, which are infrequent but potentially severe, can be mitigated. Further, the power of the TPB causal framework to predict variation in researchers’ behavioral intent provides a sound basis for interventions to increase the adoption of FH practices. Our finding that the norm and PBC were generally the main predictors of both overall and specific FH intents suggests that the wider community of bat researchers sees and accepts the need to protect bats and people and to avoid cross‐contamination of samples (leading to little variance in attitude). As expected, we observed significant differences in intent related to researchers’ career stages and the type of studies they do, but (contrary to our hypothesis) no differences related to the SES of their main fieldwork locations.

With disease researchers already required to adopt stringent biosafety practices, the main target audience for behavioral interventions is nondisease researchers. For them, as in the full model, the norm and PBC were the main predictors of general and specific FH intents. Because both the injunctive and descriptive norms of mentors were major components of the subjective norm, interventions that encourage mentors to adopt and promote FH practices, especially PPE use, should have downstream consequences for the community. Impracticality and discomfort were the key components of PBC. Discomfort is chiefly related to wearing masks and, to a lesser extent, disposable gloves. Both impracticality and discomfort may be partly alleviated by ensuring that PPE fits well, which also improves barriers to pathogen transmission (Regli et al., 2021). Indeed, the current BSG FH guidelines (Shapiro et al., 2024) now emphasize careful planning for every fieldwork activity. This may involve having all field personnel try out different sizes and types of masks (or other PPE) well in advance of fieldwork or ordering these items in various sizes.

The good‐behaviors FH construct comprises not blowing on bats and not eating, drinking, or smoking near them. Although wearing a mask precludes blowing on bats, achieving proper separation between bat research and eating and drinking requires rethinking conventional practices. Bat researchers often work at night, in challenging environments, and under intense time pressure. Hence, they traditionally treat their own biological needs as afterthoughts (e.g., opportunistically grabbing a snack). Normalizing the adoption of measures to achieve separation (designating clean and dirty areas and managing human movements and actions between these areas), as disease researchers have long been doing, will likely require instruction and resetting norms. The BSG's updated FH guidelines (Shapiro et al., 2024) provide details on how to implement these measures, but demonstrations and instruction from disease researchers and nondisease researchers who have adopted them would accelerate uptake. Opportunities to do so include workshops at professional meetings and conferences (at which T.K. has given a few such demonstrations), cross‐training, exchanges of students among laboratories, and webinars/training videos hosted on the websites of bat research organizations. Institutionally, entities charged with oversight of animal research and biosafety (e.g., Animal Care and Use Committees, Biosafety Committees) should familiarize themselves with best practice and be able to train researchers under their purview.

The role of attitude depended on researchers’ career stage. Attitude was the sole predictor of intent to use sanitary processing and PPE for late‐career (senior) researchers, who hold positive attitudes toward these behaviors. For senior researchers, these intents were not influenced by PBC or the norm, possibly reflecting a declining tendency toward social conformity with age and experience (Castrellon et al., 2024). Yet, mentors (who, we assume, include many late‐career researchers) were the most important referents for more junior (students, early‐ and mid‐career) cohorts, whose intents were largely predicated on norms and PBC. Thus, senior researchers emerge as key targets for attitudinal interventions (*sensu* Ajzen, 2006) to promote even greater uptake of FH practices within the bat research community.

We acknowledge six main study limitations. First, even though our TPB framework was effective, we tested only the specified hypotheses; there are other models of human behavior and predictors of intent that we did not evaluate. Second, to minimize intersections that might inadvertently reveal identities, we used IUCN regions as the basis for post hoc SES groupings of where respondents lived and did most of their research. However, because national income levels vary markedly within some IUCN regions, some of our SES assignments may have been imperfect. For example, we classified Oceania as high‐SES based on the number of researchers in affluent Australia, but the few researchers in the Pacific Islands might be better assigned to the lower SES group. Third, as our survey was online and only in English, non‐English speakers and individuals with reduced Internet access were presumably underrepresented in our study population. Fourth, we modeled the intent to adopt FH practices and thus measured PBC even though it may not always reflect actual behavioral control, which can influence the intent–behavior link (Ajzen, 2020). For instance, a student who believes that their project has insufficient funds to buy PPE but does not know that their advisor has money set aside for this purpose (difference between perceived and actual affordability) may spuriously point to cost as a barrier to the uptake of FH practices. Fifth, some respondents to our quantitative survey could have also taken our qualitative survey. Although we did not actively prevent participant overlap, we believe that any potential effect of nonindependence (and see van Griensven et al. [2014]) is small because our qualitative sample population was less than one tenth the size of the quantitative sample population and the overlap between questions was minimal. Finally, we did not include “honesty‐check” items in our survey (Vésteinsdóttir et al., 2019). Even though participants were instructed that their responses would be anonymized, the survey was disseminated by the IUCN BSG, which is viewed as an authoritative body, so some survey items could have been prone to social desirability bias. While acknowledging this difficulty, we took the responses at face value.

How can the universal adoption of FH practices be encouraged? Our study highlights the need to develop a community of best practice. Here, we point to the finding that authoritative bodies are key referents. Perhaps grantors could include explicit funding for FH practices or require standalone biosafety plans (akin to the data management plans required by many major funders [e.g., https://new.nsf.gov/funding/data‐management‐plan]) to help institutionalize best practice.

Other key referents were mentors. Given their influence on their junior counterparts, we propose campaigns featuring globally renowned researchers and conservation personalities wearing PPE and normalizing these behaviors and experimental tests of their efficacy. This will increase buy‐in of the entire bat research community. Studies on the use of celebrities in behavior change campaigns have mixed results in marketing generally and conservation specifically (Duthie et al., 2017; Knoll & Matthes, 2017). Critically, these studies suggest that campaign success hinges on ensuring a good fit among the endorser, the intended audience, and the behavior (Erdogan, 1999).

The IUCN SSC BSG's updated and more comprehensive FH guidelines for bat research (Shapiro et al., 2024) can serve as a blueprint for training and institutional best practice. We encourage the exploration of existing technical solutions to make PPE more practical and comfortable. For example, masks are not one‐size‐fits‐all, so researchers should try different models for the best fit. Ultimately, getting nondisease researchers to use basic PPE (masks, gloves, dedicated field clothes) and adopting best practice hinge on reducing barriers (PBC) and making these behaviors standard (the norm).

It is impossible to advance understanding of ecology, evolution, and conservation without field studies of wildlife in natural settings. Such an understanding is not only of academic and applied interest but also central to conservation. Yet, for any focal taxon (not just bats), this interface presents the risk of spillover events, which, though rare, can have devastating consequences. Hence, there is a critical need for guidelines of best practices that mitigate this risk. These guidelines should become standard operating protocol for research worldwide, given how variable the degree and nature of institutional oversight are (e.g., Institutional Animal Care and Use Committees, Natural England, the Department for Environment, Food and Rural Affairs). Still, regulatory oversight will always be limited in some regions and in the field. Therefore, a sense of personal and collective responsibility regarding FH practices must become entrenched in the wildlife research community.

## AUTHOR CONTRIBUTIONS

*Conceptualization*: Joanna L. Coleman, Ewan A. Macdonald, Tanja M. Straka, and Tigga Kingston. *Methodology*: All authors. *Validation*: All authors. *Formal analyses*: All authors. *Investigation*: Joanna L. Coleman, Ewan A. Macdonald, Tanja M. Straka, and Tigga Kingston. *Resources*: Joanna L. Coleman, Ewan A. Macdonald, Abigail L. Rutrough, Tanja M. Straka, and Tigga Kingston. *Data curation*: Joanna L. Coleman, Ewan A. Macdonald, Abigail L. Rutrough, Tanja M. Straka, and Tigga Kingston. *Writing (original draft, review, editing)*: All authors. *Visualization*: Joanna L. Coleman, Ewan A. Macdonald, Abigail L. Rutrough, and Tanja M. Straka. *Supervision*: Joanna L. Coleman, Ewan A. Macdonald, Tanja M. Straka, and Tigga Kingston. *Project administration*: Joanna L. Coleman, Ewan A. Macdonald, Tanja M. Straka, and Tigga Kingston.

## Supporting information



Supporting Information

## Data Availability

Data used for this study, including anonymized participant data, R script, and data keys, are publicly available on https://doi.org/10.6084/m9.figshare.28520129.
