# Supplementary material for: Drivers of bat researchers’ intent to adopt field hygiene practices
Source: Conserv Biol. 2026 Mar 21;40(4):e70252. doi: 10.1111/cobi.70252 (PMC13392791; doi:10.1111/cobi.70252)
Supplement: Supplementary file 1 — Supporting Information [file COBI-40-e70252-s001.docx]

**Appendix S1.** Photos of “bad behaviors”, i.e., the antithesis of field hygiene (FH) by researchers handling *Myotis lucifugus* (panel A) and *Lasiurus cinereus* (panel B). Both researchers are handling bats bare-handed, thus incurring the risk of being infected by a bat-borne pathogen—this could result from contact between bat secretions and broken skin (note the encircled faint bat-bite marks on the right thumb in panel A—the researcher had been bitten by a bat earlier that night).

The researcher in panel B is wearing a leather glove on the hand holding the bat (as is common and recommended to protect from species that bite hard, as does *L. cinereus*). However, neither person is wearing single-use nitrile gloves. Because leather gloves cannot be adequately disinfected, they can be fomites for bat-to-bat pathogens.

Researchers usually keep bats in cloth holding bags until they are done processing them and may place several conspecifics in the same bag (to avoid running out of bags). In these bags, bats often urinate and defecate, besides being in close contact—a situation that promotes bat-bat pathogen transmission.

The researcher in panel B is blowing on the bat (here, to part the fur and expose the skin for inspection). Doing so can expose bats to airborne pathogens that researchers carry. And by not wearing an N95-type mask, the researcher increases their risk of contact with bat pathogens that are airborne or with aerosolized bat secretions.


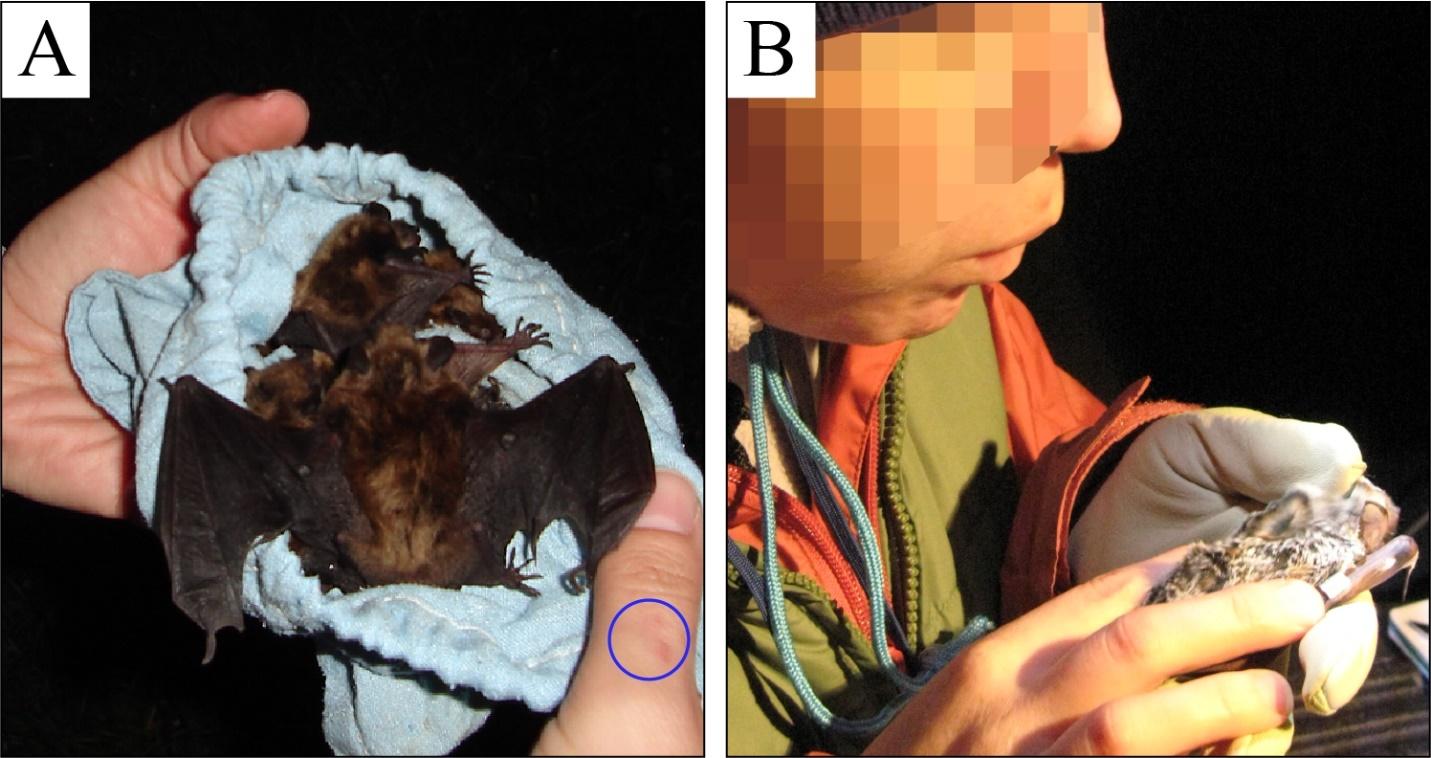
Note that these photos were taken in Canada in 2006 (panel A) and 2008 (panel B), i.e., before the development and dissemination of guidelines to protect bats from white-nose syndrome. Therefore, while the depicted behaviors were standard practice for bat researchers at the time, they are no longer acceptable.

**Appendix S2.** Qualitative survey

To identify relevant respondents—researchers who had recently led fieldwork involving close proximity (< 2m) to live, wild bats—we searched Web of Science (WoS) for all journal articles published in 2020 and 2021 with “Chiroptera” in the topics (N=539). We scanned titles, abstracts and methods sections to retain studies that clearly involved contact with live bats—this generated a pool of 290 records. To reduce geographic bias, we used WoS country-level analytics to stratify our pool and chose the first three records per represented nation. We emailed each paper’s corresponding author and asked them to complete the survey if they personally did the fieldwork in question or, if not, to pass on our request to the person who had done so.

The survey ran from 13 September through 1 November 2021, shortly after the second version of the BSG guidelines was disseminated (8 July 2021). Thus, our pool of respondents could have had two exposures to the guidelines. Both guideline roll-outs involved social-media campaigns and an email to all BSG members. The survey instrument (exported from Qualtrics) appears below.

**IUCN BSG Field hygiene questionnaire - 100921 - FINAL**

**Start of Block: PI Sheet**

Q1.1
Thank you for agreeing to participate in this research study about field hygiene practices adopted by bat researchers and handlers to mitigate the risk of disease transmission (bi-directional) between themselves and bats. This questionnaire aims to gather a set of statements that reflect the range of relevant behaviors, norms, attitudes and controls exhibited by a selected sample of bat researchers and handlers. These statements will then be used to inform a quantitative survey to be disseminated more widely.

Your participation is completely voluntary, and there are no direct benefits for your participation, but we appreciate your time and effort! You will be asked to answer 8 questions about field hygiene and then provide some demographic information. The study should only take 20 minutes to complete, and you can stop at any point and skip any questions you prefer not to answer. There are no foreseeable risks to your participation, and you can withdraw at any point, should you wish.

To protect your confidentiality, no names will be collected. Identifiers will be removed from any identifiable private information. Your information collected as part of the research will be used to support development of the quantitative study but will not be used for other studies or distributed for future research studies.

If you have any questions about this research, please contact RESEARCHER IDENTIFYING INFO REMOVED. If you have questions about your rights as a research participant, contact RESEARCHER IDENTIFYING INFO REMOVED.

I read and understood the consent form and agree to participate (1)

**End of Block: PI Sheet**

**Start of Block: Block 1: Field Hygiene**

Q2.1 In this section, we would like to hear about the field hygiene practices (procedures) that you (regularly) adopt in the field when you work with bats. In this survey, field hygiene practices refers to all measures that you take to keep yourselves and bats safe from zoonotic disease transfer while you are in contact with or close to bats (e.g. during handling, or assessing bat roosts).

Q2.2 Please briefly describe a maximum of five (5) field hygiene practices that you adopt to keep yourself and bats safe. The order is not important.

- 1 (1) ________________________________________________
- 2 (2) ________________________________________________
- 3 (3) ________________________________________________
- 4 (4) ________________________________________________
- 5 (5) ________________________________________________

*Carry Forward Entered Choices - Entered Text from "Please briefly describe a maximum of five (5) field hygiene practices that you adopt to keep yourself and bats safe. The order is not important. "*

|  |
| --- |

Q2.4 For how many years have you been using these field hygiene practices? (please enter a number)

- 1 (1) ________________________________________________
- 2 (2) ________________________________________________
- 3 (3) ________________________________________________
- 4 (4) ________________________________________________
- 5 (5) ________________________________________________

Q2.3 In one or two sentences, please share your opinion about the value of the field hygiene practices you use.

Q2.5 Where did you learn about the field hygiene practices you use?

Q2.6 What makes it easy for you to adopt the field hygiene practices you use?

Q2.7 What makes it difficult for you to adopt the field hygiene practices you use?

Q2.8 Are there any field hygiene practices that you would like to use but that you currently can not? If so, please describe the practice(s) and state why you are unable to use it (them).

Q2.9 Who determines which field hygiene practices you currently use?

**End of Block: Block 1: Field Hygiene**

**Start of Block: Block 2 Demography**

Q3.1 In this section we wish to find out a little bit more about you.

Q3.2 Please share your gender identity in the box below. You can leave this blank if you would rather not say.

Q3.3 What is your age in years?

Q3.4 Which of the following best describes your current career stage?

- Undergraduate student (1)
- Graduate student (2)
- Early career (within 10 years of final graduate degree) (3)
- Mid career (10-25 years since final degree) (4)
- Late career (> 25 years since final degree) (5)
- Retired (6)
- Other (7) ________________________________________________

Q3.5 Which of the following best describes your current or most recent employer?

- University / research institution (1)
- Non-profit organisation (2)
- Government or state organisation or agency (3)
- Consultancy (4)
- Other (5) ________________________________________________

Q3.6 Where is your primary residence at the moment? (you may only select one response)

- Northern Africa (1)
- Sub-Saharan Africa (2)
- Central Asia (3)
- South Asia (4)
- East Asia (5)
- Southeast Asia (6)
- Australasia/Oceania (7)
- Middle East (8)
- North America (9)
- Central America (10)
- South America (11)
- Western Europe (12)
- Eastern Europe (13)
- Other (14) ________________________________________________

Q3.7 Where, primarily, do you conduct your research at the moment? (you may select more than one response)

- Northern Africa (1)
- Sub-Saharan Africa (2)
- Central Asia (3)
- South Asia (4)
- East Asia (5)
- Southeast Asia (6)
- Australasia/Oceania (7)
- Middle East (8)
- North America (9)
- Central America (10)
- South America (11)
- Western Europe (12)
- Eastern Europe (13)
- Other (14) ________________________________________________

**End of Block: Block 2 Demography**

**Start of Block: Block 3**

Q4.1 Please type your name in the box below. The only purpose for collecting this information is to avoid duplicate entries and to spare you unnecessary reminder emails from us - rest assured that we will delete the columns with these data before we look at the associated responses.

________________________________________________________________

**End of Block: Block 3**

**Appendix S3.** Quantitative survey

We performed a pilot survey (n=45 respondents) in August 2022 and made minor changes to items that were problematic. We set Qualtrics parameters to ensure a fully anonymised dataset. The full survey ran 13 September 2022 through 7 February 2023, with recruitment via social media and email lists and reminders, especially to participants from countries with low response rates.

**Participant Information Sheet:**

IUCN Bat Specialist Group Field Hygiene Questionnaire 2022

Thank you for agreeing to participate in this research study about field hygiene practices adopted by bat researchers and handlers to mitigate the risk of disease transmission (bi-directional) between themselves and bats. This questionnaire aims to gather information about a range of relevant behaviours, norms, attitudes and controls exhibited by bat researchers and handlers. The purpose of this study is to understand how widely best practices are adopted and the barriers to the adoption of these best practices.

Your participation is completely voluntary, and there are no direct benefits for your participation, but we appreciate your time and effort! You will be asked to answer a series of questions about field hygiene and then provide some demographic information. The study should only take 15-20 minutes to complete, and you can stop at any point and skip any questions you prefer not to answer. There are no foreseeable risks to your participation, and you can withdraw at any point, should you wish.

To protect your confidentiality, no names will be collected. Identifiers will be removed from any identifiable private information. Your information collected as part of the research will be used to support development of policies and campaigns intended to promote field hygiene best practices, findings may also be published in relevant academic journals.

At the end of this questionnaire, you will be presented with another, one-question survey, which you may choose to complete if you want to be entered into a raffle (draw) to win one, copy of the book "Handbook of the Mammals of the World – Volume 9 Bats". This one question will ask for your email address, but your response will be treated separately from your response to this questionnaire - thus preserving data anonymity.

If you have any questions about this research, please contact RESEARCHER IDENTIFYING INFO REMOVED FOR MS PEER REVIEW. If you have questions about your rights as a research participant, contact RESEARCHER IDENTIFYING INFO REMOVED FOR MS PEER REVIEW.

- I read and understood the consent form and agree to participate

**Introduction:**

The purpose of this questionnaire is to discover the reasons why researchers who perform bat **fieldwork** do or do not adopt various **field hygiene (FH)** practices. By **fieldwork**, we mean “the practice by which investigators seek out organisms in their natural habitats to collect samples and associated materials, perform experiments and record natural-history observations” - this definition is from [Ramírez-Castañeda et al (2022)](https://www.pnas.org/doi/abs/10.1073/pnas.2122667119) A set of principles and practical suggestions for equitable fieldwork in biology.

**Definition of field hygiene practices**

The IUCN BSG follows Cunningham et al. 2003* and defines field hygiene as **“a set of best practices using standard, simple measures to minimize the risk that research activities result in pathogen pollution, the human-facilitated moving or transferring of pathogens between species and sites”. Briefly, these guidelines include:**

- **Avoid contact when possible**
- **Wear a face covering**
- **Do not blow on bats**
- **Wash and disinfect hands before starting work and at the end of work**
- **Use nitrile or latex gloves (and change / disinfect regularly)**
- **Avoid touching your face**
- **Disinfect equipment**
- **Get relevant vaccines**

**For full guidelines, please** [click here](https://www.iucnbsg.org/uploads/6/5/0/9/6509077/amp_recommendations_for_researchers_final.pdf)**.**

*Cunningham AA, Daszak P, Rodriguez JP. Pathogen pollution: defining a parasitological threat to biodiversity conservation. J Parasitol. 2003;89(Suppl):S78-83.

**Instructions:**

**General guidelines to complete this survey**

Many questions in this survey make use of rating scales with seven (7) places; you are to choose the number that best describes your opinion. For example, if you were asked to rate “The weather in Paris” on such a scale, the 7 places should be interpreted as follows:


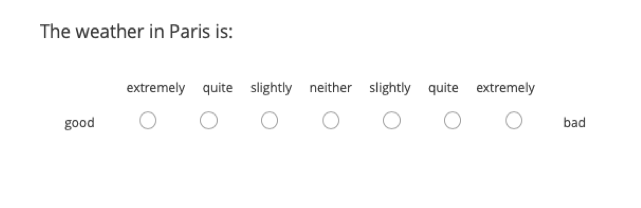


If you think the weather in Paris is slightly good, then you would choose the third button (from the left), as follows:


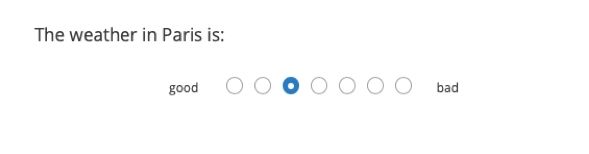


If you think the weather in Paris is extremely good, then you would choose the first button (from the left), as follows:


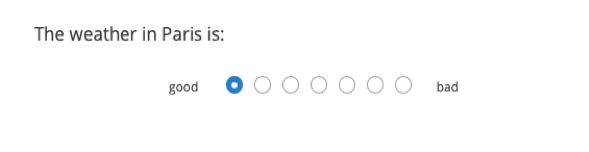


If you think the weather in Paris is quite bad, then you would choose the sixth button (from the left), as follows:


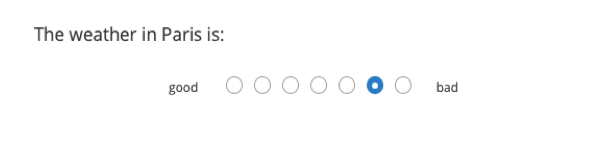


If you think the weather in Paris is neither good nor bad, then you would choose the middle button, as follows:


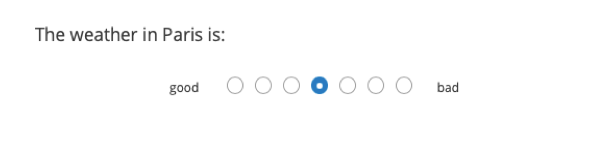


Please be aware that scales may be oriented from **positive to negative** or the other way around (left to right). Meaning, the positions of ‘good’ and ‘bad’ may be reversed. As such, please pay close attention when making your selection.

Also, if possible, please complete this survey on a desktop computer rather than a handheld electronic device, i.e., mobile phone.

**Field Hygiene Practices:**

**Field hygiene practices and you**

We wish to highlight that our ultimate goal is simply to improve the guidelines and enhance the uptake of field hygiene (FH) practices by understanding what researchers are actually doing (i.e., what constitutes best practice, and what researchers want to see in the guidelines). As such, we really want your honest, candid answers. There is zero “penalty” to saying that you do not adopt various behaviors.

Q1_CURR_RES: Which of the following best describes the type of research that you will do next time you are working with bats in the field?

- Analysis of diet
- Disease-related
- Diversity surveys
- Natural history / demographics
- Parasites
- Other __________________________________________________

Q2_BIOL_SAMPLES: Next time you are working with bats in the field, do you plan to collect biological samples from bats?

- Yes (1)
- Unsure (2)
- No (3)

**Direct Antecedents of Behaviour:**

Q3_INTENT: I intend to adopt the full set of field hygiene (FH) practices next time I am working with bats in the field.

| Extremely unlikely |  |  |  |  |  |  |  | Extremely likely |
| --- | --- | --- | --- | --- | --- | --- | --- | --- |

Q4_INTENT_ATT: In general, adopting FH practices next time I am working with bats in the field is…

| Good |  |  |  |  |  |  |  | Bad |
| --- | --- | --- | --- | --- | --- | --- | --- | --- |

Q5_INTENT_INORM: Most people whose opinions I value think that I should adopt FH practices while working with bats.

| Definitely false |  |  |  |  |  |  |  | Definitely true |
| --- | --- | --- | --- | --- | --- | --- | --- | --- |

Q6_INTENT_DNORM: Most people whose opinions I value will adopt FH practices next time they are working with bats in the field.

| Definitely true |  |  |  |  |  |  |  | Definitely false |
| --- | --- | --- | --- | --- | --- | --- | --- | --- |

Q7_INTENT_PBC: It is **my decision** whether or not I adopt FH practices next time I am working with bats in the field.

| Strongly disagree |  |  |  |  |  |  |  | Strongly agree |
| --- | --- | --- | --- | --- | --- | --- | --- | --- |

To view the full set of IUCN Field Hygiene guidelines please [click here](https://www.iucnbsg.org/uploads/6/5/0/9/6509077/amp_recommendations_for_researchers_final.pdf).

**Attitudes Couplets:**

Q8_DIS_H2B_BEL: My adoption of field hygiene (FH practices) next time I am working with bats in the field will protect **bats** from potential pathogens that **I may carry**.

| Strongly agree |  |  |  |  |  |  |  | Strongly disagree |
| --- | --- | --- | --- | --- | --- | --- | --- | --- |

Q9_DIS_H2B_OUTC: Protecting **bats** from potential pathogens that **I may carry** is…

| Not important |  |  |  |  |  |  |  | Very important |
| --- | --- | --- | --- | --- | --- | --- | --- | --- |

Q10_DIS_B2H_BEL: My adoption of FH practices next time I am working with bats in the field will protect **me** from potential pathogens that **bats may carry**.

| Strongly agree |  |  |  |  |  |  |  | Strongly disagree |
| --- | --- | --- | --- | --- | --- | --- | --- | --- |

Q11_DIS_B2H_OUTC: Protecting **myself**from potential pathogens that **bats may carry** is…

| Not important |  |  |  |  |  |  |  | Very important |
| --- | --- | --- | --- | --- | --- | --- | --- | --- |

Q12_DIS_B2B_BEL: My adoption of FH practices next time I am working with bats in the field will protect **bats**from potential pathogens that **other bats may carry**.

| Strongly agree |  |  |  |  |  |  |  | Strongly disagree |
| --- | --- | --- | --- | --- | --- | --- | --- | --- |

Q13_DIS_B2B_OUTC: Protecting **bats**from potential pathogens that **other bats** **may carry** is…

| Not important |  |  |  |  |  |  |  | Very important |
| --- | --- | --- | --- | --- | --- | --- | --- | --- |

Q14_XCONTAM_BEL: My adoption of FH practices next time I am working with bats in the field will **minimize cross-contamination** of any samples (e.g., DNA contamination) I collect from bats.

| Strongly agree |  |  |  |  |  |  |  | Strongly disagree |
| --- | --- | --- | --- | --- | --- | --- | --- | --- |

Q15_XCONTAM_OUTC: Minimizing cross-contamination of any samples I collect from bats is…

| Not important |  |  |  |  |  |  |  | Very important |
| --- | --- | --- | --- | --- | --- | --- | --- | --- |

To view the full set of IUCN Field Hygiene guidelines please [click here](https://www.iucnbsg.org/uploads/6/5/0/9/6509077/amp_recommendations_for_researchers_final.pdf).

**Norms: Authoritative body:**

**These next three (3) questions ask you to think about whichever authoritative body (or bodies) sets/set the guidelines or requirements for your research.**

Q16_NORM_NAME_AUTH: To your knowledge, what is/are the authoritative body/bodies that sets/set the guidelines or requirements for your research? If you cannot name the authoritative body/bodies, then you may key in "I do not know" or "I am unsure".

[text entry box:] ________________________________________________________________

Q17_INORM_AUTH_BEL: The relevant authoritative body/bodies expects/expect that I will adopt field hygiene (FH) practices next time I am working with bats in the field.

| Extremely likely |  |  |  |  |  |  |  | Extremely unlikely |
| --- | --- | --- | --- | --- | --- | --- | --- | --- |

Q18_INORM_MENT_MOTIV: In general, I want to do what the relevant authoritative body/bodies says I should do.

| Strongly agree |  |  |  |  |  |  |  | Strongly disagree |
| --- | --- | --- | --- | --- | --- | --- | --- | --- |

To view the full set of IUCN Field Hygiene guidelines please [click here](https://www.iucnbsg.org/uploads/6/5/0/9/6509077/amp_recommendations_for_researchers_final.pdf).

**Norms: Mentor:**

**These next four (4) questions ask you to think about your mentor. Please do not overthink this – we just mean the first mentor that comes to mind (e.g., lab leader, supervisor, boss, person who trained you, etc.).**

Q19_INORM_BEL_MENT: My mentor expects that I will adopt field hygiene (FH) practices next time I am working with bats in the field.

| Extremely likely |  |  |  |  |  |  |  | Extremely unlikely |
| --- | --- | --- | --- | --- | --- | --- | --- | --- |

Q20_INORM_MOTIV_MENT: In general, I want to do what my mentor thinks I should do.

| Strongly agree |  |  |  |  |  |  |  | Strongly disagree |
| --- | --- | --- | --- | --- | --- | --- | --- | --- |

Q21_DNORM_BEL_MENT: My mentor adopts FH practices when working with bats in the field.

| Completely true |  |  |  |  |  |  |  | Completely false |
| --- | --- | --- | --- | --- | --- | --- | --- | --- |

Q22_DNORM_MOTI_MENT: When it comes to fieldwork, I want to emulate (be like) my mentor.

| Very much |  |  |  |  |  |  |  | Not at all |
| --- | --- | --- | --- | --- | --- | --- | --- | --- |

To view the full set of IUCN Field Hygiene guidelines please [click here](https://www.iucnbsg.org/uploads/6/5/0/9/6509077/amp_recommendations_for_researchers_final.pdf).

**Norms: Peers:**

**These next four (4) questions ask you to think about your peers who also conduct fieldwork on bats. Please do not overthink this – we just mean, for example, fellow bat researchers, colleagues, etc.**

Q23_INORM_BEL_PEER: My peers expect that I will adopt (field hygiene) FH practices next time I am working with bats in the field.

| Extremely likely |  |  |  |  |  |  |  | Extremely unlikely |
| --- | --- | --- | --- | --- | --- | --- | --- | --- |

Q24_INORM_MOTI_PEER: In general, I want to do what my peers think I should do.

| Strongly agree |  |  |  |  |  |  |  | Strongly disagree |
| --- | --- | --- | --- | --- | --- | --- | --- | --- |

Q25_DNORM_BEL_PEER: My peers adopt FH practices when they are working with bats in the field.

| Completely true |  |  |  |  |  |  |  | Completely false |
| --- | --- | --- | --- | --- | --- | --- | --- | --- |

Q26_DNORM_MOTI_PEER: When it comes to field work, I want to emulate (be like) my peers.

| Very much |  |  |  |  |  |  |  | Not at all |
| --- | --- | --- | --- | --- | --- | --- | --- | --- |

To view the full set of IUCN Field Hygiene guidelines please [click here](https://www.iucnbsg.org/uploads/6/5/0/9/6509077/amp_recommendations_for_researchers_final.pdf).

**Perceived Behavioral Control:**

Q27_PBC_BEL_PRAC: Adopting field hygiene (FH) practices will make bat fieldwork difficult (for example, eyeglasses fogging up, loss of manual dexterity, lots of gear to manage/dispose of).

| Definitely true |  |  |  |  |  |  |  | Definitely false |
| --- | --- | --- | --- | --- | --- | --- | --- | --- |

Q28_PBC_POWER_PRAC: Finding fieldwork difficult will deter me from adopting FH practices next time I am working with bats in the field.

| Strongly agree |  |  |  |  |  |  |  | Strongly disagree |
| --- | --- | --- | --- | --- | --- | --- | --- | --- |

Q29_PBC_BEL_DISC: I will experience physical discomfort when adopting FH practices for bat fieldwork.

| Extremely likely |  |  |  |  |  |  |  | Extremely unlikely |
| --- | --- | --- | --- | --- | --- | --- | --- | --- |

Q30_PBC_POWER_DISC: Experiencing physical discomfort will deter me from adopting FH practices next time I am working with bats in the field.

| Strongly agree |  |  |  |  |  |  |  | Strongly disagree |
| --- | --- | --- | --- | --- | --- | --- | --- | --- |

Q31_PBC_BEL_$: I can financially afford any items needed for FH practices for bat fieldwork.

| Definitely false |  |  |  |  |  |  |  | Definitely true |
| --- | --- | --- | --- | --- | --- | --- | --- | --- |

Q32_PBC_POWER_$: Being able to afford these items will enable me to adopt FH practices next time I am working with bats in the field.

| Strongly agree |  |  |  |  |  |  |  | Strongly disagree |
| --- | --- | --- | --- | --- | --- | --- | --- | --- |

Q33_PBC_BEL_ACQ: I can get whatever items I need to adopt FH practices when I am doing fieldwork with bats (for example, items are accessible / available in / deliverable in my location).

| Easily |  |  |  |  |  |  |  | With difficulty |
| --- | --- | --- | --- | --- | --- | --- | --- | --- |

Q34_PBC_POWER_ACQ: Being able to get these items will enable me to adopt FH practices next time I am working with bats in the field.

| Strongly agree |  |  |  |  |  |  |  | Strongly disagree |
| --- | --- | --- | --- | --- | --- | --- | --- | --- |

To view the full set of IUCN Field Hygiene guidelines please [click here](https://www.iucnbsg.org/uploads/6/5/0/9/6509077/amp_recommendations_for_researchers_final.pdf).

**Specific Intent:**

**This section asks about your specific field hygiene practices.**

Q35_SPEC_INTENT: **Next time you work with bats in the field, how likely (on a scale of 1 to 7, where 1 = extremely likely and 7 = extremely unlikely) are you to do each of the following?**

|  | 1 | 2 | 3 | 4 | 5 | 6 | 7 |
| --- | --- | --- | --- | --- | --- | --- | --- |
| Wear disposable (nitrile) gloves when handling bats and tissues: |  |  |  |  |  |  |  |
| Wear protective (e.g., leather) gloves when handling bats and tissues: |  |  |  |  |  |  |  |
| Wear an N95-type mask when in proximity (< 2 m) to bats: |  |  |  |  |  |  |  |
| Disinfect tools / instruments / surfaces: |  |  |  |  |  |  |  |
| Put only one bat in each bat bag: |  |  |  |  |  |  |  |
| Clean bat bags between each use: |  |  |  |  |  |  |  |
| Blow on bats (e.g., to get them to stop biting, part their fur to look for ectoparasites): |  |  |  |  |  |  |  |
| Eat or drink while handling bats (i.e., during the interaction): |  |  |  |  |  |  |  |
| Minimize team size (no. of people in the field): |  |  |  |  |  |  |  |
| Use dedicated field clothes (items I will not wear for other occasions): |  |  |  |  |  |  |  |
| Be fully vaccinated against COVID-19 (vaccine series plus booster): |  |  |  |  |  |  |  |
| Protect myself against rabies (e.g. pre-exposure vaccine, booster, titre check, etc.) |  |  |  |  |  |  |  |
| Get a post-exposure rabies vaccine (e.g., if I am bitten by a bat): |  |  |  |  |  |  |  |

Q36_COMMENTS: Do you have any comments about the adoption of field hygiene (FH) practices that you wish to share?

________________________________________________________________

________________________________________________________________

________________________________________________________________

________________________________________________________________

To view the full set of IUCN Field Hygiene guidelines please [click here](https://www.iucnbsg.org/uploads/6/5/0/9/6509077/amp_recommendations_for_researchers_final.pdf).

**Demographics:**

**A bit more about you**

Q37_GENDER: How do you describe yourself (select as many as appropriate)?

- Man
- Woman
- Non-binary / third gender
- Prefer to self-describe

__________________________________________________

- Prefer to not answer

Q38_AGE: What is your age in years? You may choose not to answer.

________________________________________________________________

Q39_CAREER_STAGE: Which of the following best describes your current career stage?

- Undergraduate student
- Graduate student
- Early career (within 10 years of final graduate degree)
- Mid career (10-25 years since final degree)
- Late career / senior position (>25 years since final degree)
- Retired
- Other __________________________________________________

Q40_EMPLOYER: Which of the following best describes your current or most recent employer? (You may only select one option.)

- University / research institution
- Non-profit organisation
- Government or state organization or agency
- Consultancy
- Other __________________________________________________

Q41_PRIM_RESID: Where is your primary residence? (You may only select one option.)

- Northern Africa
- Sub-Saharan Africa
- Central Asia
- South Asia
- East Asia
- Southeast Asia
- Australasia/Oceania
- Middle East
- North America
- Central America
- South America
- Western Europe
- Eastern Europe
- Other __________________________________________________

Q42_RES_LOCATION: Where, primarily, do you conduct your research? (You may select multiple options.)

- Northern Africa
- Sub-Saharan Africa
- Central Asia
- South Asia
- East Asia
- Southeast Asia
- Australasia/Oceania
- Middle East
- North America
- Central America
- South America
- Western Europe
- Eastern Europe
- Other __________________________________________________

Q43_BAT_TYPES: Which of the following taxa do you work with? (You may select multiple options.)

- Pteropodidae
- All other bats without Pteropodidae
- Bats in general
- Other mammals: __________________________________________________

Q44_PAST_RES: In the past five years, my main bat-related research questions have been:

- Analysis of diet
- Disease-related
- Diversity surveys
- Natural history / demographics
- Parasites
- Other __________________________________________________

**Thanks:**

Thank you for participating in our survey. If you wish to be take part in the prize draw for the 2019 book *Handbook of the Mammals of the World, 9. Bats*, please click the blue "next" arrow. This will take you to an entirely separate survey where we will collect your contact information. This contact information will be kept completely separate from your survey responses and will be deleted as soon as the prize draw has been completed. If you do not wish to be entered into the prize draw, then you can simply close this window.

**Appendix S4.** Distributions of responses to survey questions and correlation matrices of conceptual couplets

This section graphically presents the raw data relating to the conceptual “couplets” used in our SEM analyses. The SEM analyses combine couplets of items because they are intended to capture a belief and the influence of that belief on the same underlying construct. Each plot in this document therefore corresponds to a couplet used in the SEM, and we would therefore expect to find positive significant correlations throughout.

---------------------------------------------------------------------------------------------------------------------
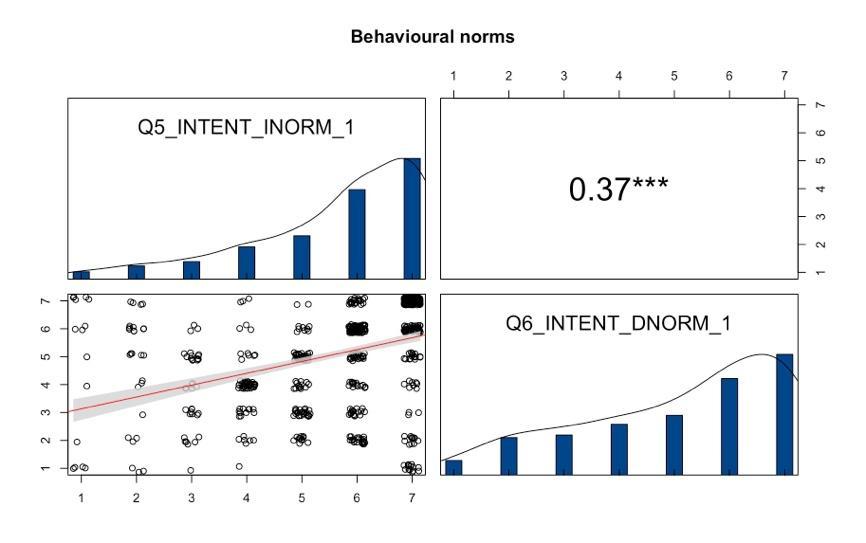


Behavioral norms (overall):

Q5_INTENT_INORM: Most people whose opinions I value think that I should adopt FH practices while working with bats. [*7 point scale; Definitely false::Definitely true*]

Q6_INTENT_DNORM: Most people whose opinions I value will adopt FH practices next time they are working with bats in the field. [*7 point scale; Definitely false::Definitely true*]

---------------------------------------------------------------------------------------------------------------------

Behavioral attitude:

Protect bats from people


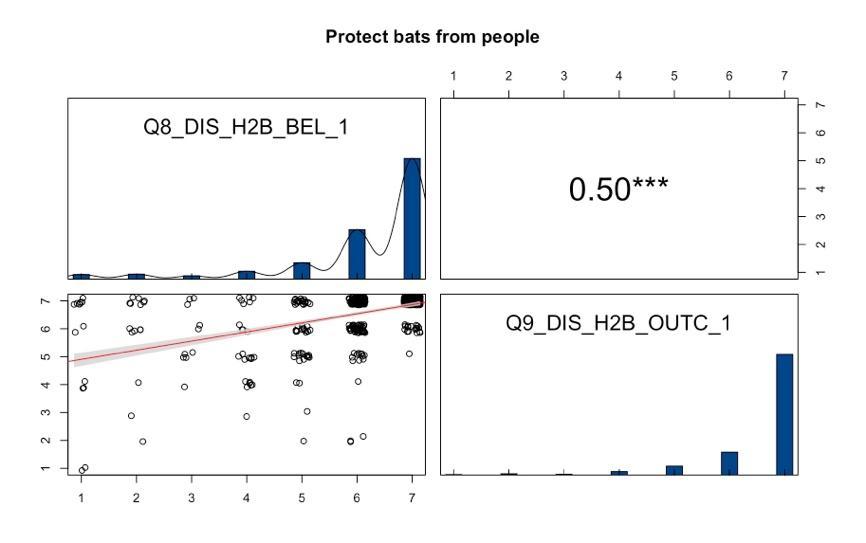
Q8_DIS_H2B_BEL: My adoption of field hygiene (FH practices) next time I am working with bats in the field will protect bats from potential pathogens that I may carry. [*7 point scale; Strongly disagree::Strongly agree*]

Q9_DIS_H2B_OUTC: Protecting bats from potential pathogens that I may carry is. . . [*7 point scale; Not important::Very important*]

We see that respondents are almost unanimous in believing that adopting FH practices is important but are slightly more equivocal about whether doing so will protect bats from people.

---------------------------------------------------------------------------------------------------------------------

Protect people from bats
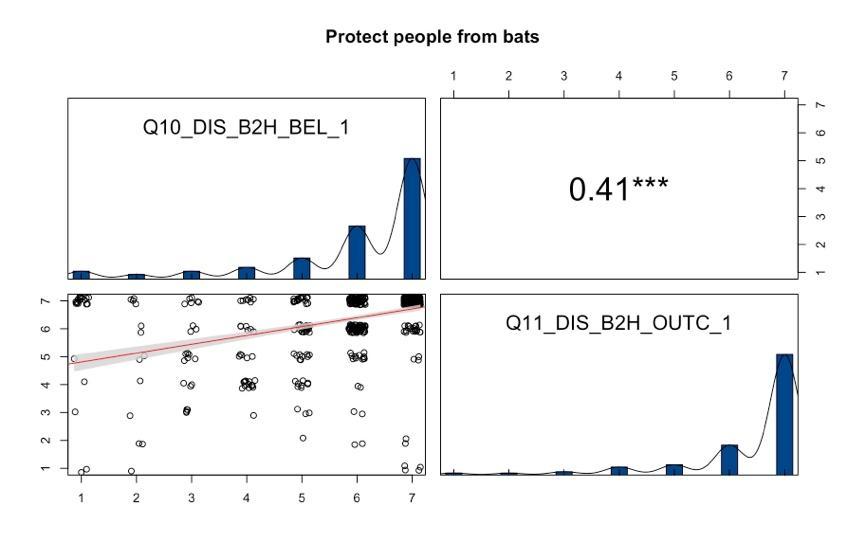


Q10_DIS_B2H_BEL: My adoption of FH practices next time I am working with bats in the field will protect me from potential pathogens that bats may carry. [*7 point scale; Strongly disagree::Strongly agree*]

Q11_DIS_B2H_OUTC: Protecting myself from potential pathogens that bats may carry is. . . [*7 point scale; Not important::Very important*]

We see that respondents are almost unanimous in believing that adopting FH practices is important but are slightly more equivocal about whether doing so will protect people from bats.

---------------------------------------------------------------------------------------------------------------------

Protect bats from bats

Q12_DIS_B2B_BEL: My adoption of FH practices next time I am working with bats in the field will protect bats from potential pathogens that other bats may carry. [*7 point scale; Strongly disagree::Strongly agree*]
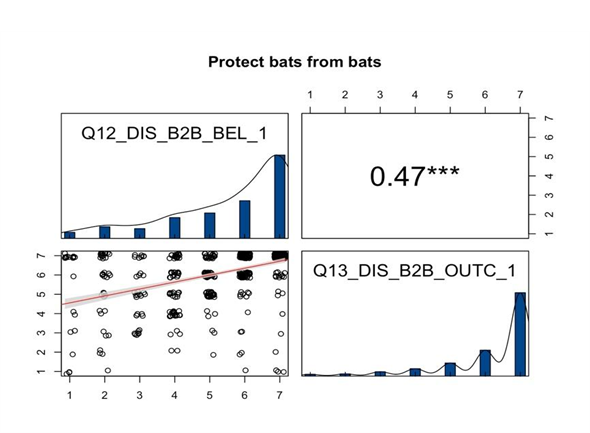


Q13_DIS_B2B_OUTC: Protecting bats from potential pathogens that other bats may carry is. . . [*7 point scale; Not important::Very important*]

We see that respondents are almost unanimous in believing that adopting FH practices is important but that they are slightly more equivocal about whether doing so will protect bats from bats.

---------------------------------------------------------------------------------------------------------------------

No cross contamination


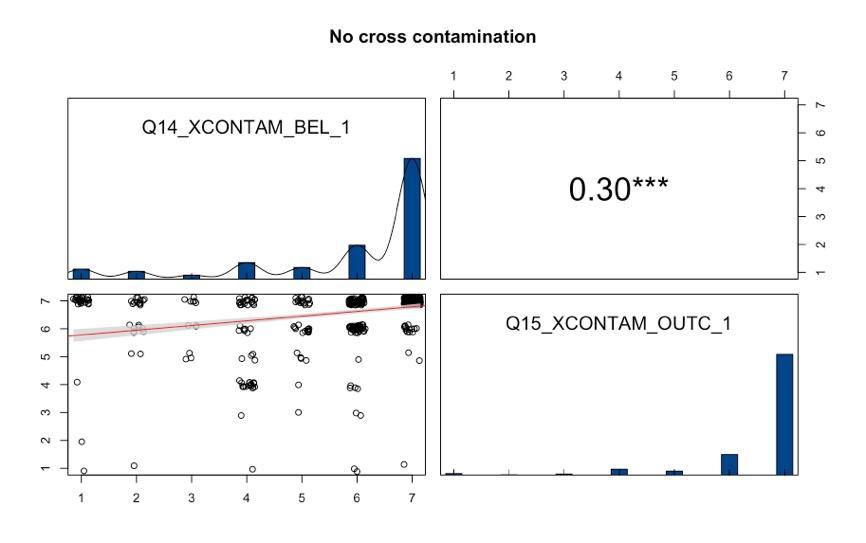
Q14_XCONTAM_BEL: My adoption of FH practices next time I am working with bats in the field will minimize cross-contamination of any samples (e.g., DNA contamination) I collect from bats. [*7 point scale; Strongly disagree::Strongly agree*]

Q15_XCONTAM_OUTC: Minimizing cross-contamination of any samples I collect from bats is. . . [*7 point scale; Not important::Very important*]

We see that respondents are almost unanimous in believing that adopting FH practices is important and believe that doing so will reduce cross contamination.

---------------------------------------------------------------------------------------------------------------------

Norm

Authoritative body:

Q17_INORM_AUTH_BEL: The relevant authoritative body/bodies expects/expect that I will adopt field hygiene (FH) practices next time I am working with bats in the field. [*7 point scale; Extremely unlikely::Extremely likely*]
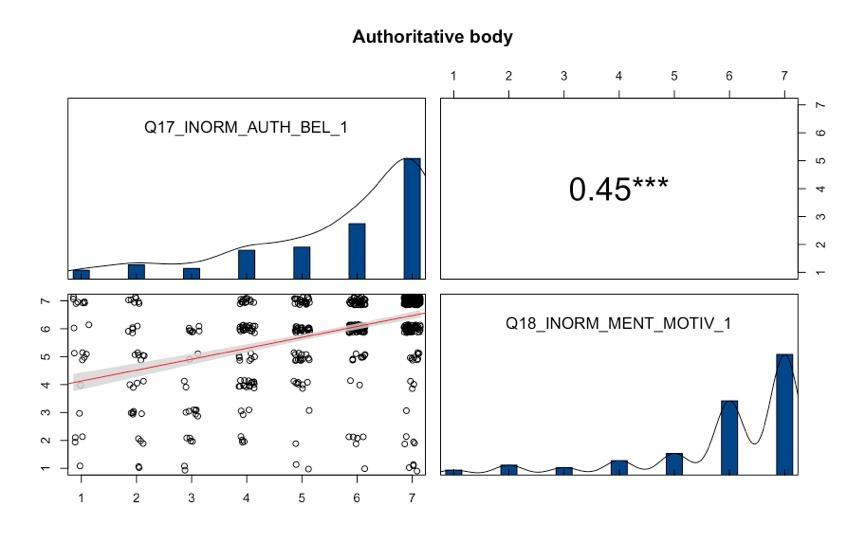


Q18_INORM_MENT_MOTIV: In general, I want to do what the relevant authoritative body/bodies says I should do. [*7 point scale; Strongly disagree::Strongly agree*]

Desire to do what the authoritative body wants us to do seems stronger than the belief that the authoritative body expects people to adopt FH practices.

---------------------------------------------------------------------------------------------------------------------


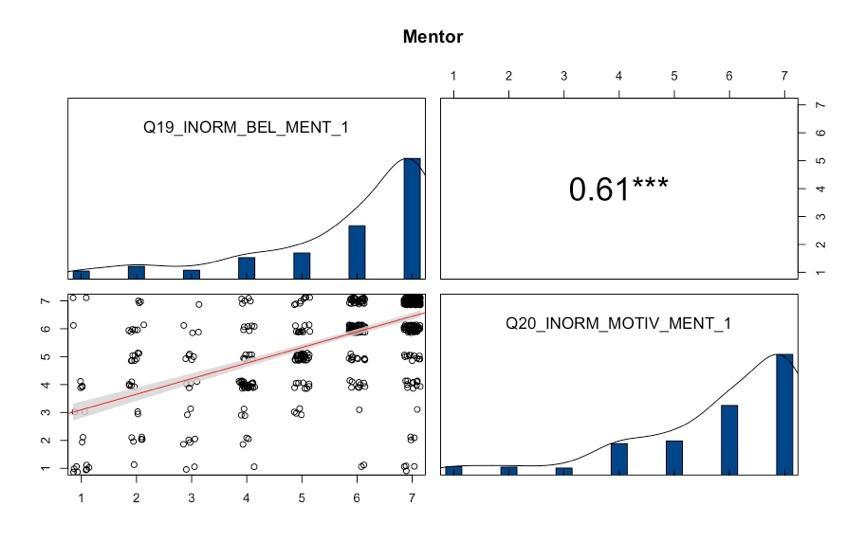
Mentor (injunctive norm)

Q19_INORM_BEL_MENT: My mentor expects that I will adopt field hygiene (FH) practices next time I am working with bats in the field. [*7 point scale; Extremely unlikely::Extremely likely*]

Q20_INORM_MOTIV_MENT: In general, I want to do what my mentor thinks I should do. [*7 point scale; Strongly disagree::Strongly agree*]

---------------------------------------------------------------------------------------------------------------------
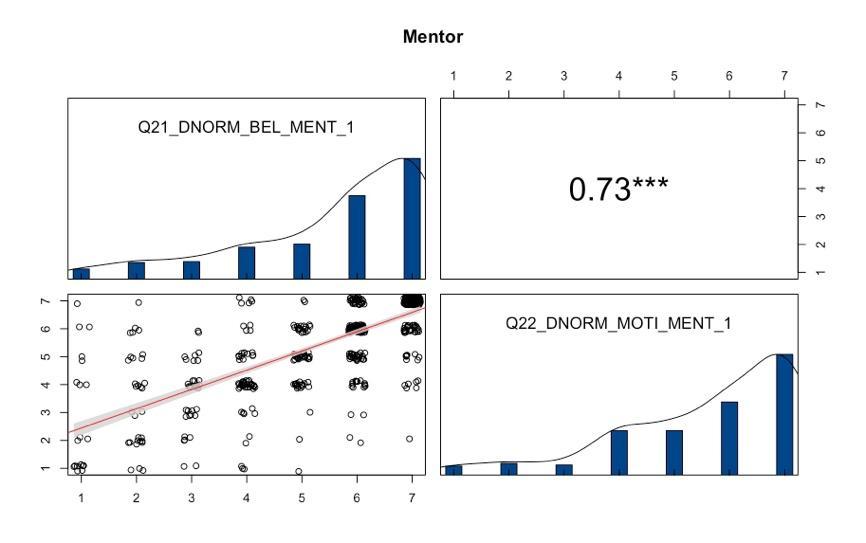


Mentor (descriptive norm)

Q21_DNORM_BEL_MENT: My mentor adopts FH practices when working with bats in the field. [*7 point scale; completely false::Completely true*]

Q22_DNORM_MOTI_MENT: When it comes to fieldwork, I want to emulate (be like) my mentor. [*7 point scale; Not at all::Very much*]

---------------------------------------------------------------------------------------------------------------------

Mentor (injunctive vs descriptive norm)
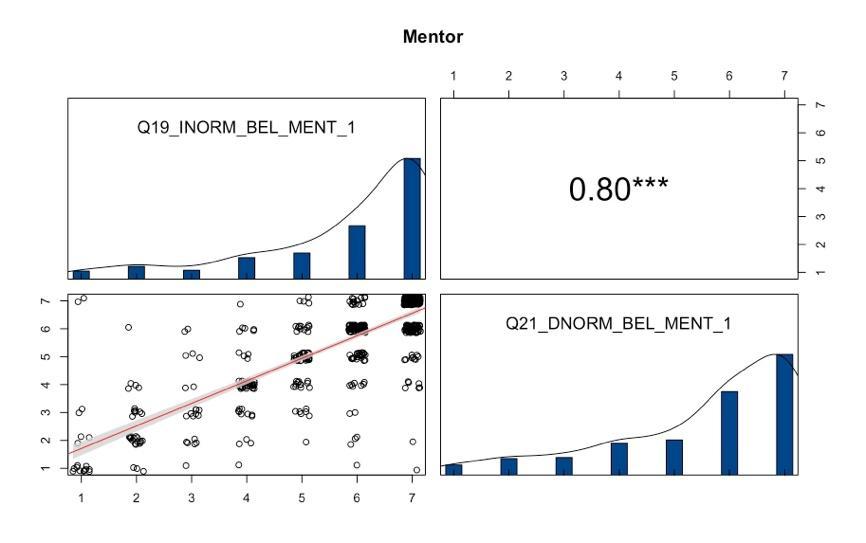


Q19_INORM_BEL_MENT: My mentor expects that I will adopt field hygiene (FH) practices next time I am working with bats in the field. [*7 point scale; Extremely unlikely::Extremely likely*]

Q21_DNORM_BEL_MENT: My mentor adopts FH practices when working with bats in the field. [*7 point scale; completely false::Completely true*]

---------------------------------------------------------------------------------------------------------------------

Peers (injunctive norm)
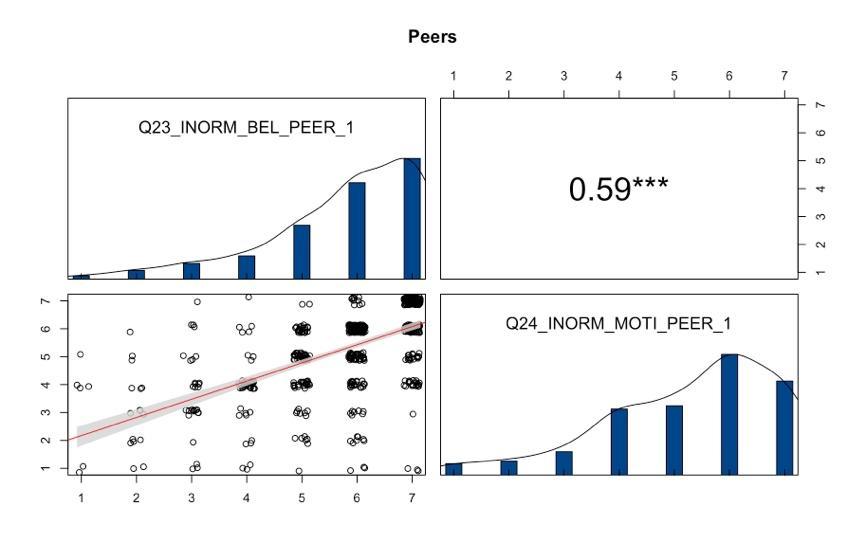


Q23_INORM_BEL_PEER: My peers expect that I will adopt (field hygiene) FH practices next time I am working with bats in the field. [*7 point scale; Extremely unlikely::Extremely likely*]

Q24_INORM_MOTI_PEER: In general, I want to do what my peers think I should do. [*7 point scale; Strongly disagree::Strongly agree*]

---------------------------------------------------------------------------------------------------------------------
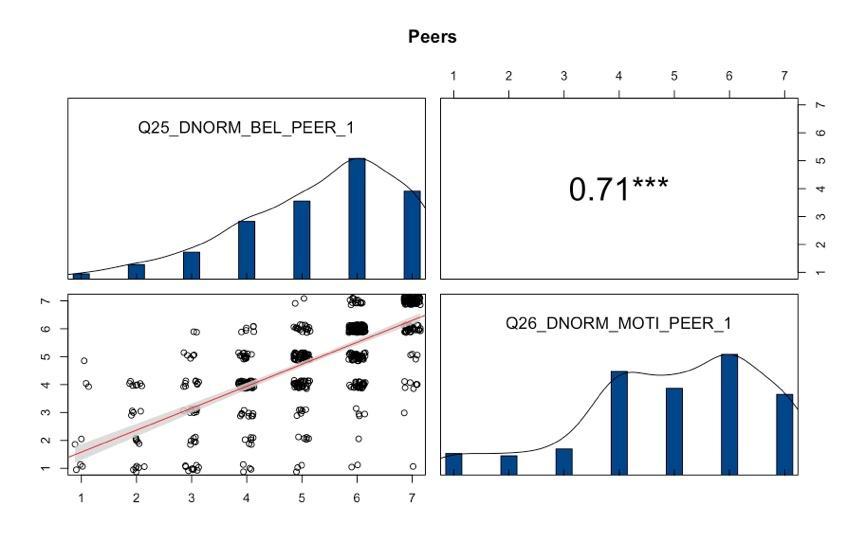


Peers (descriptive norm)

Q25_DNORM_BEL_PEER: My peers adopt FH practices when they are working with bats in the field. [*7 point scale; completely false::Completely true*]

Q26_DNORM_MOTI_PEER: When it comes to field work, I want to emulate (be like) my peers. [*7 point scale; Not at all::Very much*]

---------------------------------------------------------------------------------------------------------------------
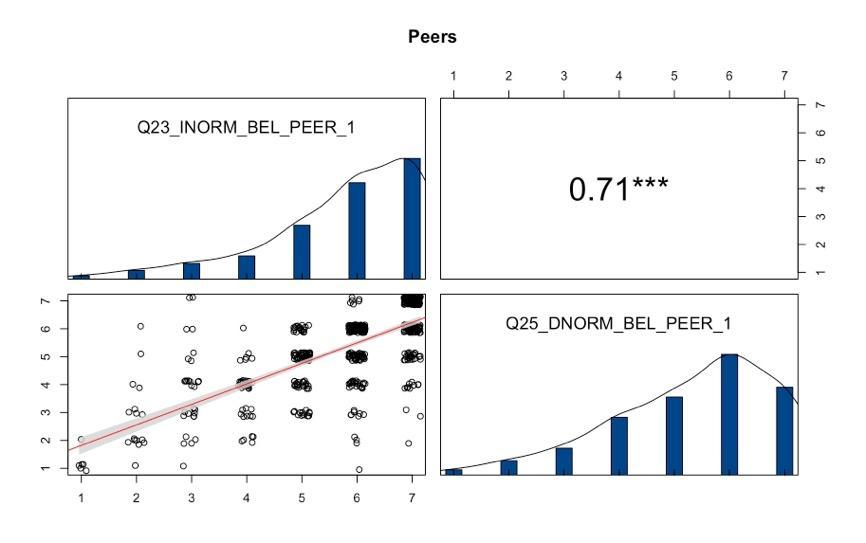


Peers (injunctive vs descriptive norm)

Q23_INORM_BEL_PEER: My peers expect that I will adopt (field hygiene) FH practices next time I am working with bats in the field. [*7 point scale; Extremely unlikely::Extremely likely*]

Q25_DNORM_BEL_PEER: My peers adopt FH practices when they are working with bats in the field. [*7 point scale; completely false::Completely true*]

---------------------------------------------------------------------------------------------------------------------

Perceived behavioral control

Practicality:

Q27_PBC_BEL_PRAC: Adopting field hygiene (FH) practices will make bat fieldwork difficult (for example, eyeglasses fogging up, loss of manual dexterity, lots of gear to manage/dispose of). [*7 point scale; Definitely false::Definitely true*]

Q28_PBC_POWER_PRAC: Finding fieldwork difficult will deter me from adopting FH practices next time I am working with bats in the field. [*7 point scale; Strongly disagree::Strongly agree*]


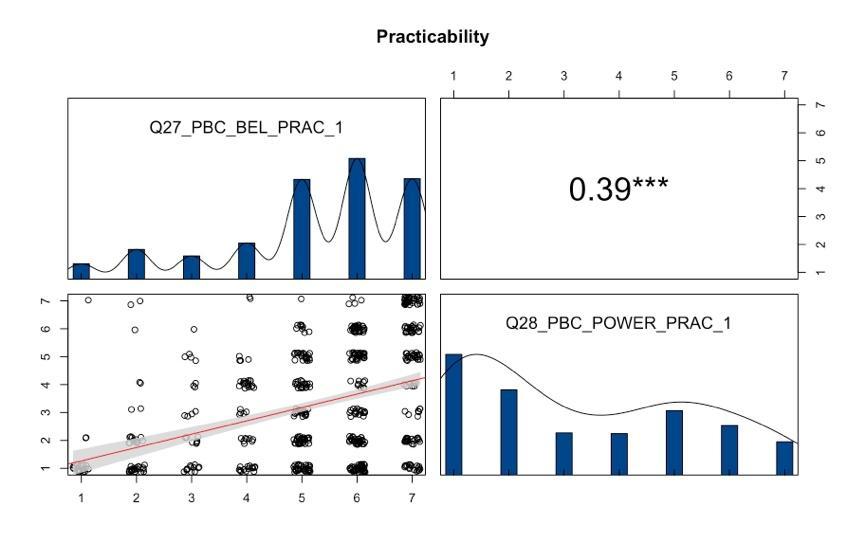


NOTE. Although these two items are positively correlated, Q28_PBC_POWER_PRAC appears strongly weighted towards lower scores. This is because this item asks if practical difficulties would deter bat researchers from adopting FH practices, and our respondents are hard to deter.

---------------------------------------------------------------------------------------------------------------------


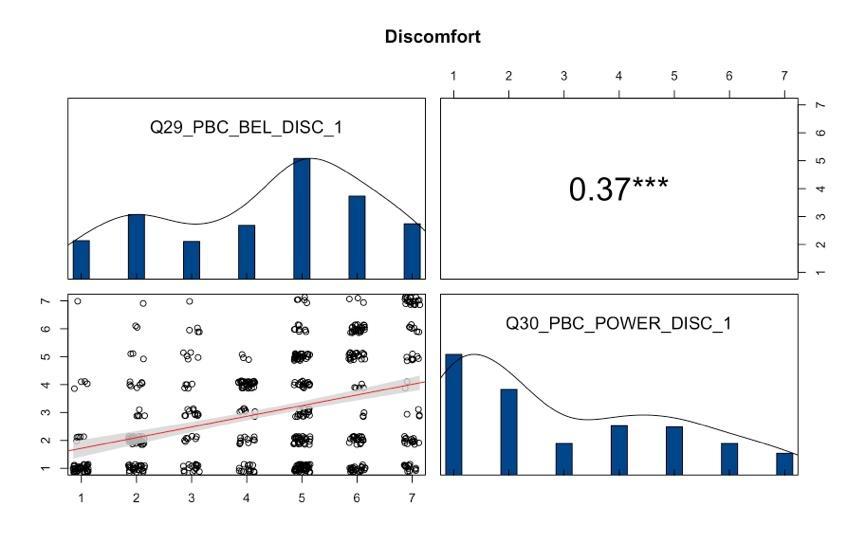
Discomfort:

Q29_PBC_BEL_DISC: I will experience physical discomfort when adopting FH practices for bat fieldwork. [*7 point scale; Extremely unlikely::Extremely likely*]

Q30_PBC_POWER_DISC: Experiencing physical discomfort will deter me from adopting FH practices next time I am working with bats in the field. [*7 point scale; Strongly disagree::Strongly agree*]

NOTE. Although these two items are positively correlated, Q30_PBC_POWER_DISC appears strongly weighted towards lower scores. This is because this item asks if discomfort would deter bat researchers from adopting FH practices, and our respondents are hard to deter.

Financial:

Q31_PBC_BEL_FIN: I can financially afford any items needed for FH practices for bat fieldwork. [*7 point scale; Definitely false::Definitely true*]


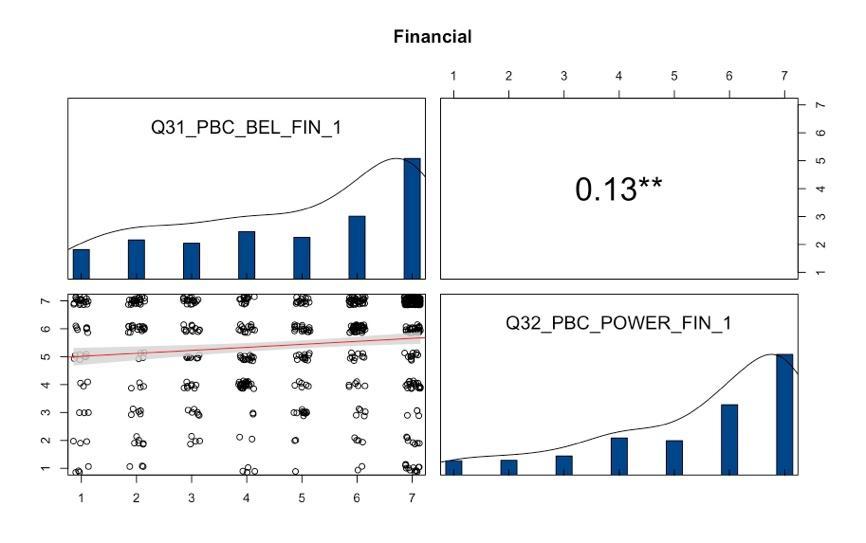
Q32_PBC_POWER_FIN: Being able to afford these items will enable me to adopt FH practices next time I am working with bats in the field. [*7 point scale; Strongly disagree::Strongly agree*]

---------------------------------------------------------------------------------------------------------------------


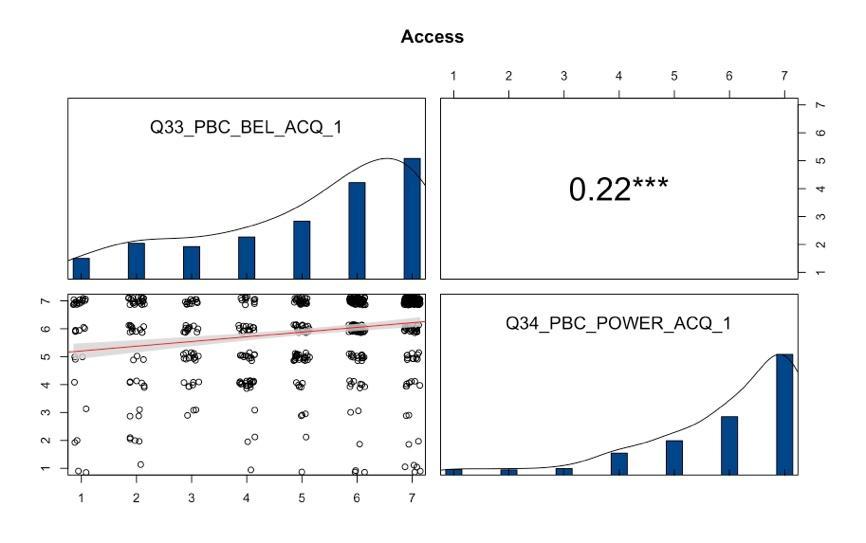
Access:

Q33_PBC_BEL_ACQ: I can get whatever items I need to adopt FH practices when I am doing fieldwork with bats (for example, items are accessible / available in / deliverable in my location). [*7 point scale; With difficulty::Easily*]

Q34_PBC_POWER_ACQ: Being able to get these items will enable me to adopt FH practices next time I am working with bats in the field. [*7 point scale; Strongly disagree::Strongly agree*]

---------------------------------------------------------------------------------------------------------------------

Control:
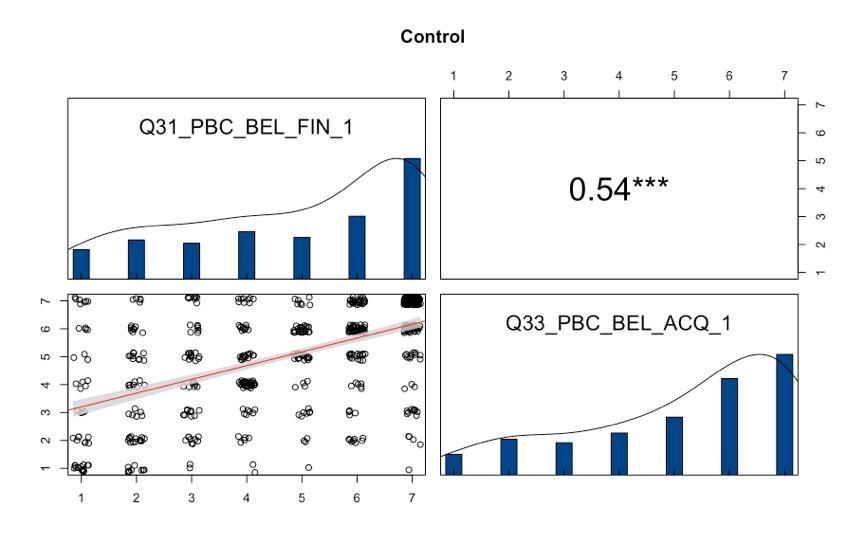


Q31_PBC_BEL_FIN: I can financially afford any items needed for FH practices for bat fieldwork. [*7 point scale; Definitely false::Definitely true*]

Q33_PBC_BEL_ACQ: I can get whatever items I need to adopt FH practices when I am doing fieldwork with bats (for example, items are accessible / available in / deliverable in my location). [*7 point scale; With difficulty::Easily*]

---------------------------------------------------------------------------------------------------------------------

Q32_PBC_POWER_FIN: Being able to afford these items will enable me to adopt FH practices next time I am working with bats in the field. [*7 point scale; Strongly disagree::Strongly agree*]


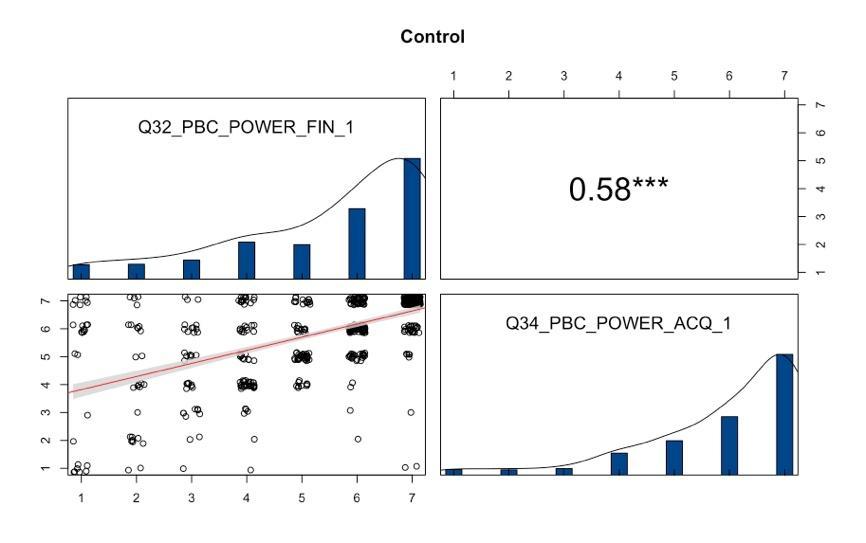
Q34_PBC_POWER_ACQ: Being able to get these items will enable me to adopt FH practices next time I am working with bats in the field. [*7 point scale; Strongly disagree::Strongly agree*]

**Appendix S5.** Summarized responses to demographic questions

Q37_GENDER: How do you describe yourself (select as many as appropriate)?
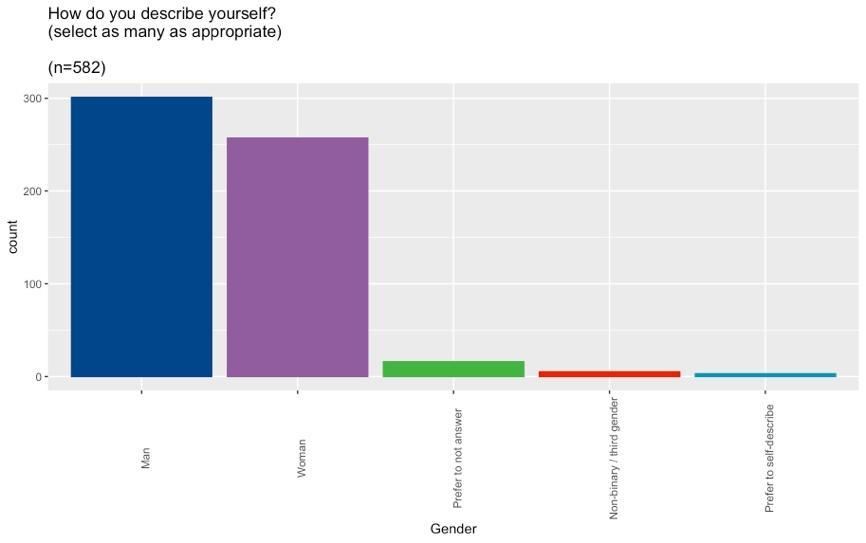


N = 582 (417 NAs)

| **Gender** | **Count** | **%** |
| --- | --- | --- |
| Man | 301 | 51.72 |
| Woman | 257 | 44.16 |
| Prefer to not answer | 16 | 2.75 |
| Non-binary / third gender | 5 | 0.86 |
| Prefer to self-describe | 3 | 0.52 |

Note. Respondents were able to select as many responses as appropriate, but no respondents provided multiple responses.

Q38_AGE What is your age in years? You may choose not to answer.

N = 494 (505 NAs)

3 people entered values >100 suggesting they failed to answer this question correctly.

For the people who answered correctly, our sample has a range from 20 to 78 years with a median age of 38 years.


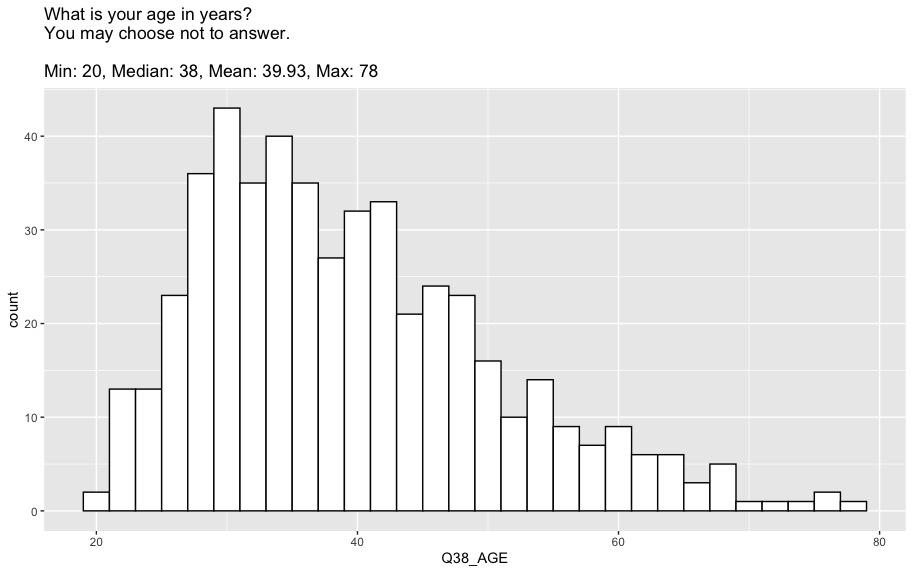

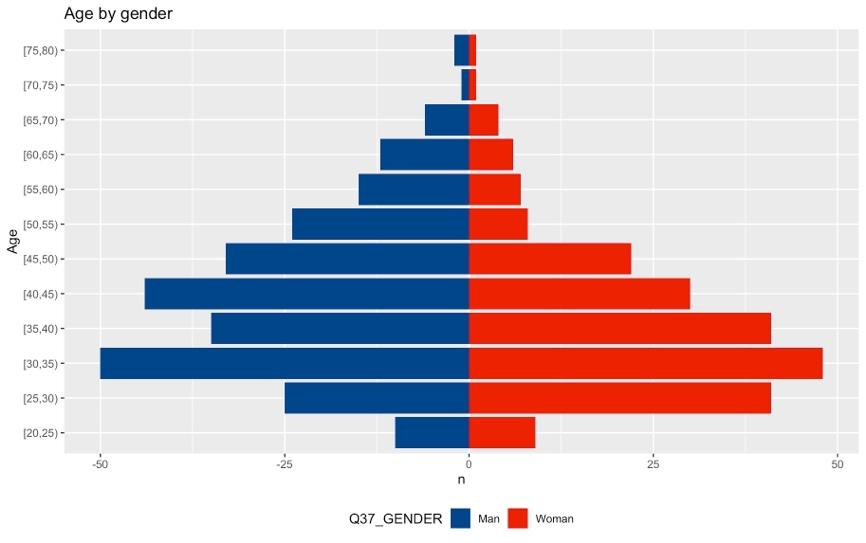


Interestingly, the age pyramids (at right above), which only include respondents who identified as either men or women n=475), reveal that men in our sample are typically older than women are.

| **Gender** | **n** | **mean** | **SD** | **median** | **min** | **max** | **range** |
| --- | --- | --- | --- | --- | --- | --- | --- |
| Man | 257 | 41.42 | 11.45 | 40 | 21 | 78 | 57 |
| Woman | 218 | 37.9 | 10.62 | 35 | 20 | 77 | 57 |

Q39_CAREER_STAGE Which of the following best describes your current career stage?

N = 583 (416 NAs)

| **Career Stage** | **Count** | **%** |
| --- | --- | --- |
| Early career (within 10 years of final graduate degree) | 183 | 31.39 |
| Mid career (10-25 years since final degree) | 166 | 28.47 |
| Graduate student | 99 | 16.98 |
| Late career / senior position (>25 years since final degree) | 76 | 13.04 |
| Other | 26 | 4.46 |
| Undergraduate student | 19 | 3.26 |
| Retired | 14 | 2.4 |


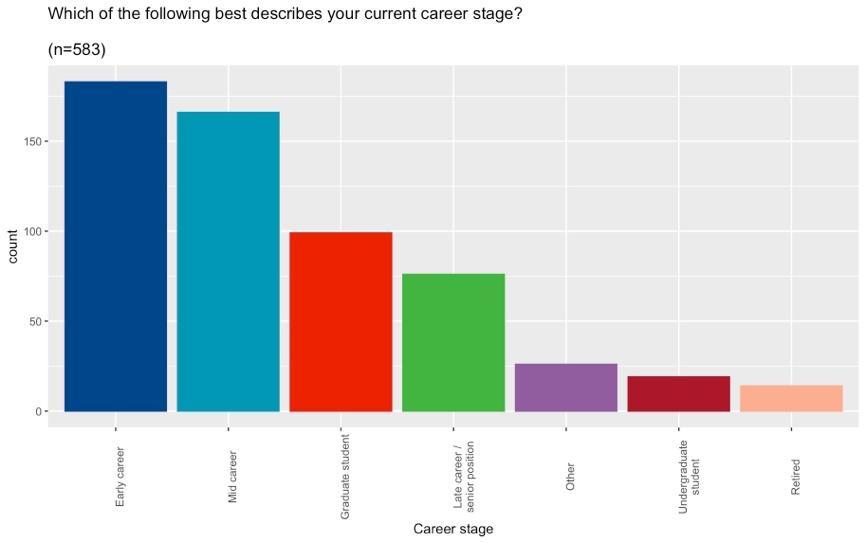


Q40_EMPLOYER Which of the following best describes your current or most recent employer? (You may only select one option.)

N= 581 (418 NAs)
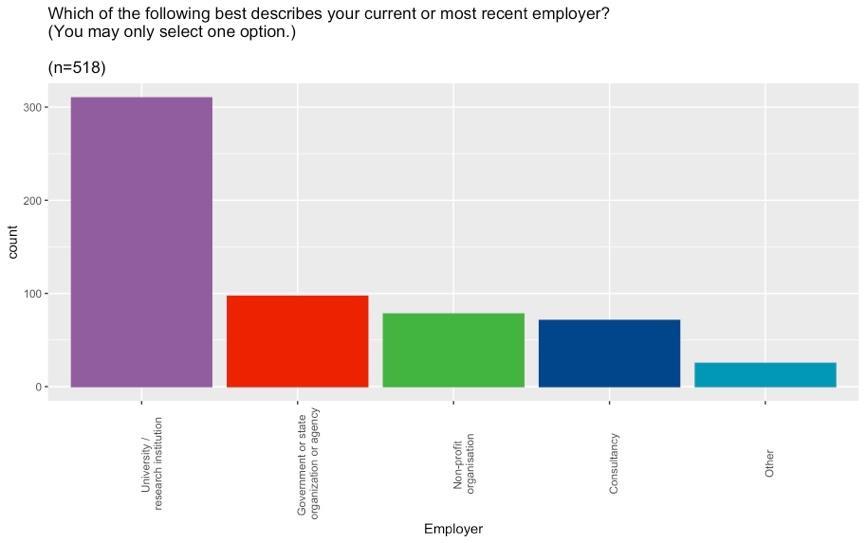


| **Employer** | **count** | **%** |
| --- | --- | --- |
| University / research institution | 310 | 53.36 |
| Government or state organization or agency | 97 | 16.7 |
| Non-profit organisation | 78 | 13.43 |
| Consultancy | 71 | 12.22 |
| Other | 25 | 4.3 |

Q41_PRIM_RESID Where is your primary residence? (You may only select one option.)

N= 584 (415 NAs)
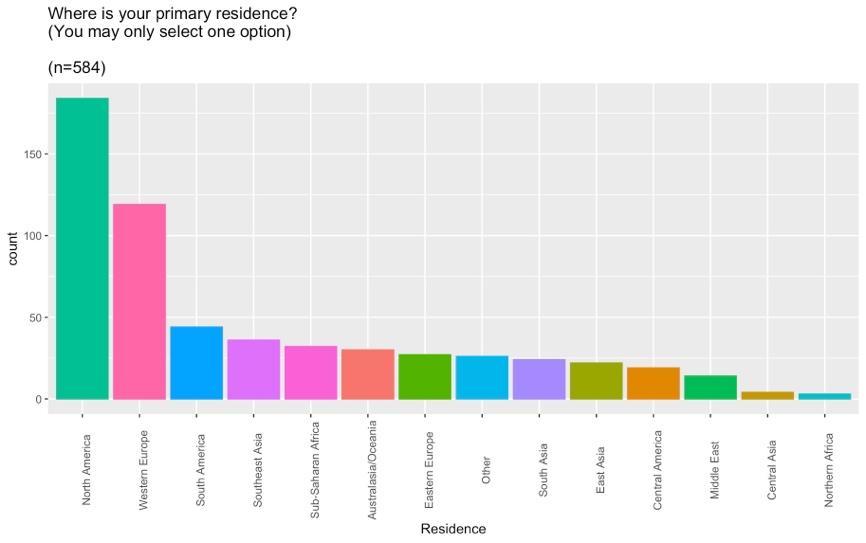


| **Primary Residence** | **count** | **%** |
| --- | --- | --- |
| North America | 184 | 31.51 |
| Western Europe | 119 | 20.38 |
| South America | 44 | 7.53 |
| Southeast Asia | 36 | 6.16 |
| Sub-Saharan Africa | 32 | 5.48 |
| Australasia/Oceania | 30 | 5.14 |
| Eastern Europe | 27 | 4.62 |
| Other | 26 | 4.45 |
| South Asia | 24 | 4.11 |
| East Asia | 22 | 3.77 |
| Central America | 19 | 3.25 |
| Middle East | 14 | 2.4 |
| Central Asia | 4 | 0.68 |
| Northern Africa | 3 | 0.51 |

Q42_RES_LOCATION Where, primarily, do you conduct your research? (You may select multiple options.)

N= 582, 417 NAs


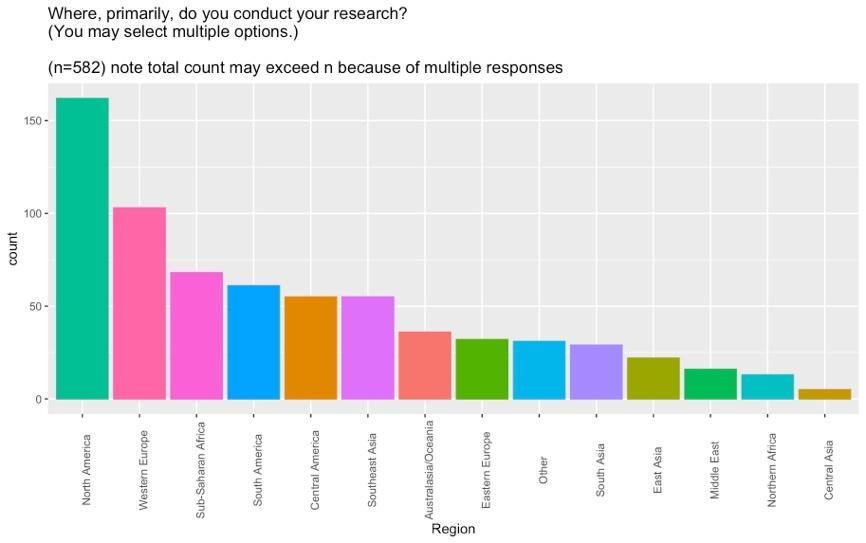


| **Research Location** | **count** |
| --- | --- |
| North America | 162 |
| Western Europe | 103 |
| Sub-Saharan Africa | 68 |
| South America | 61 |
| Southeast Asia | 55 |
| Central America | 55 |
| Australasia/Oceania | 36 |
| Eastern Europe | 32 |
| Other | 31 |
| South Asia | 29 |
| East Asia | 22 |
| Middle East | 16 |
| Northern Africa | 13 |
| Central Asia | 5 |

Proportion of respondents selecting each response was not calculated as participants could select >1 response

Q43_BAT_TYPES Which of the following taxa do you work with? (You may select multiple options.)

N= 582 417 NAs
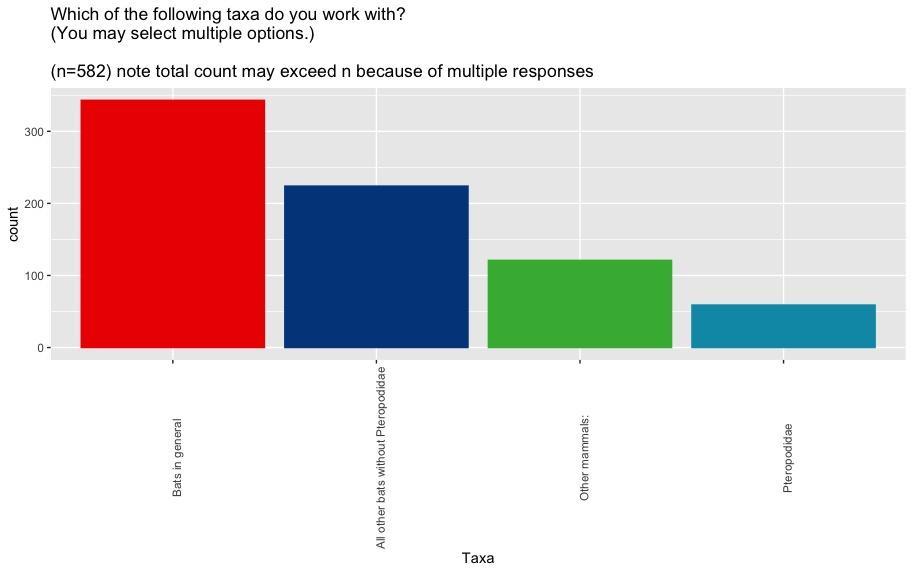


| **Bat Types** | **count** |
| --- | --- |
| Bats in general | 343 |
| All other bats without Pteropodidae | 224 |
| Other mammals | 121 |
| Pteropodidae | 59 |

Proportion of respondents selecting each response was not calculated as participants could select >1 response

121 participants selected “other mammals” with 67 providing a textual response.

Q44_PAST_RES In the past five years, my main bat-related research questions have been:

N= 581 418NAs
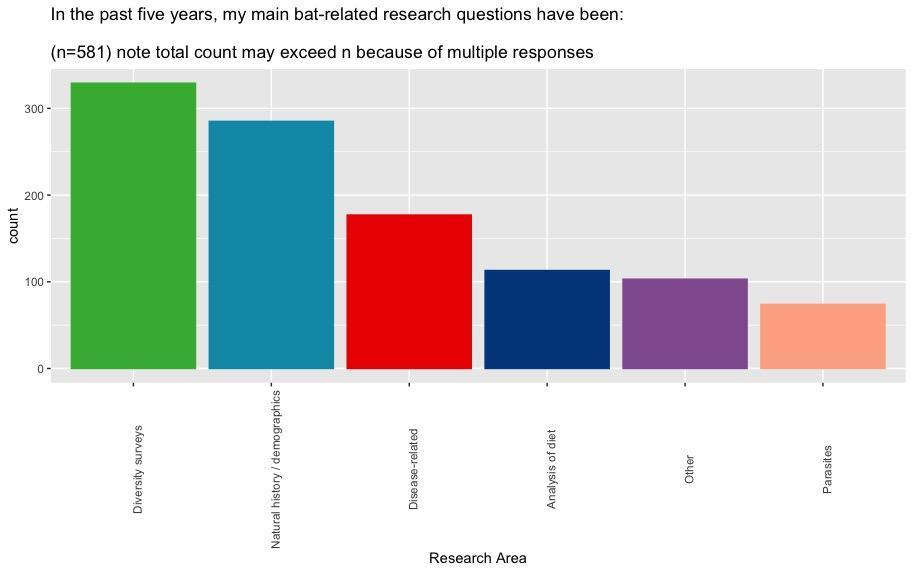


| **Research areas** | **count** |
| --- | --- |
| Diversity surveys | 329 |
| Natural history / demographics | 285 |
| Disease-related | 177 |
| Analysis of diet | 113 |
| Other | 103 |
| Parasites | 74 |

Proportion of respondents selecting each response was not calculated as participants could select >1 response. 103 participants selected “Other” with 84 providing a textual response.

**Appendix S6. Summary statistics of the full field hygiene data set**

General intent to adopt field hygiene was high, with a mean (Likert) score of 5.72 (on a scale from 1-7).

Table S6. Summary statistics of the full field hygiene data set.

| **Indicator** | **Mean** | **SD** | **Skewness** | **Kurtosis** |
| --- | --- | --- | --- | --- |
| Intent General | 5.715 | 1.615 | -1.417 | 1.177 |
| Protect people from bats | 6.388 | 0.981 | -2.114 | 4.947 |
| Protect bats from people | 6.177 | 1.145 | -1.584 | 2.172 |
| Protect bats from bats | 5.856 | 1.306 | -1.099 | 0.614 |
| Minimize cross-contamination | 6.267 | 1.103 | -1.539 | 1.758 |
| Authority figure | 5.778 | 1.351 | -1.158 | 0.584 |
| Mentor: injunctive norm | 5.743 | 1.438 | -1.245 | 0.96 |
| Mentor: descriptive norm | 5.552 | 1.52 | -1.025 | 0.278 |
| Peers: injunctive norm | 5.518 | 1.295 | -0.864 | 0.238 |
| Peers: descriptive norm | 5.134 | 1.408 | -0.621 | -0.204 |
| Impracticality | 4.237 | 1.391 | -0.121 | -0.228 |
| Discomfort | 3.669 | 1.4 | 0.173 | -0.122 |
| Financial | 2.836 | 1.321 | 0.433 | -0.14 |
| Access | 2.487 | 1.18 | 0.599 | 0.075 |
| Disinfect tools | 6.18 | 1.436 | -2.034 | 3.594 |
| One bat per bag | 6.344 | 1.483 | -2.466 | 5.17 |
| Clean bags between uses | 5.91 | 1.726 | -1.584 | 1.402 |
| Blow on bats | 3.299 | 2.244 | 0.395 | -1.371 |
| Eat/drink around bats | 1.906 | 1.748 | 1.977 | 2.615 |
| Disposable gloves | 6.119 | 1.611 | -2.006 | 3.03 |
| N95 type masks | 5.454 | 1.889 | -1.056 | -0.118 |
| Dedicated field clothes | 5.174 | 2.021 | -0.873 | -0.573 |
| Protective gloves | 5.933 | 1.755 | -1.677 | 1.632 |
| Covid-19 | 6.514 | 1.377 | -3.114 | 8.712 |
| Rabies pre-exposure | 6.442 | 1.39 | -2.813 | 7.15 |
| Rabies post-exposure | 5.344 | 2.107 | -0.967 | -0.518 |

| **Appendix S7. Invariance testing for the full and multigroup regression models**.  We evaluated and confirmed weak and strong measurement invariance for all constructs across multigroup models of: career stage (N=552); research type (N=565), and socioeconomic status (SES) of fieldwork location (N=570). Partial strong invariance was only required for the SES of fieldwork location group (Table S9), where impracticality, discomfort, and dedicated field clothes were variant (Table S8).  Table S7. Model fit and invariance testing for multigroup models. | | | | | | | | | | |
| --- | --- | --- | --- | --- | --- | --- | --- | --- | --- | --- |
| **Model** | **χ2** | **df** | **p** | **CFI** | **TLI** | **SRMR** | **RMSEA** | **RMSEA 90% CI** | **Δ CFI** | **Pass?** |
| *Career Stage* |  |  |  |  |  |  |  |  |  |  |
| Configural | 1668.572 | 1068 | <0.001 | 0.889 | 0.864 | 0.0719 | 0.0638 | 0.0579 - 0.0697 | — | — |
| Weak | 1741.951 | 1119 | <0.001 | 0.885 | 0.866 | 0.0772 | 0.0635 | 0.0577 - 0.0692 | 0.00415 | Yes |
| Strong | 1804.099 | 1173 | <0.001 | 0.883 | 0.87 | 0.079 | 0.0624 | 0.0567 - 0.0681 | 0.00151 | Yes |
| *SES* |  |  |  |  |  |  |  |  |  |  |
| Configural | 1317.797 | 801 | <0.001 | 0.904 | 0.884 | 0.0642 | 0.0583 | 0.0526 - 0.0638 | — | — |
| Weak | 1377.023 | 835 | <0.001 | 0.9 | 0.883 | 0.0679 | 0.0585 | 0.0529 - 0.0639 | 0.00467 | Yes |
| Strong | 1541.463 | 871 | <0.001 | 0.876 | 0.861 | 0.0735 | 0.0637 | 0.0584 - 0.0688 | 0.02380 | No |
| Partial | 1462.862 | 868 | <0.001 | 0.89 | 0.876 | 0.0698 | 0.0601 | 0.0547 - 0.0654 | 0.00978 | Yes |
| *Research Type* |  |  |  |  |  |  |  |  |  |  |
| Configural | 1379.913 | 801 | <0.001 | 0.892 | 0.869 | 0.0656 | 0.0619 | 0.0564 - 0.0674 | — | — |
| Weak | 1439.460 | 835 | <0.001 | 0.888 | 0.869 | 0.0676 | 0.062 | 0.0566 - 0.0674 | 0.00475 | Yes |
| Strong | 1527.253 | 871 | <0.001 | 0.878 | 0.863 | 0.0699 | 0.0633 | 0.058 - 0.0685 | 0.00963 | Yes |

| **Appendix S8. Latent parameter testing for the full and multigroup regression models.**  Table S8. Latent parameter testing for full and multi-group models. | | | | | | | | | |
| --- | --- | --- | --- | --- | --- | --- | --- | --- | --- |
| Model | *χ^2^* | *df* | *CFI* | *TLI* | *SRMR* | *RMSEA* | Δ *χ^2^* | Δ *df* | *p* |
| *Full Model* |  |  |  |  |  |  |  |  |  |
| Structural | 628.517 | 267 | 0.932 | 0.918 | 0.0485 | 0.0451 | — | — | — |
| Pruned | 633.836 | 271 | 0.932 | 0.919 | 0.0490 | 0.0448 | 5.319 | 4 | 0.256 |
| *Career Stage* |  |  |  |  |  |  |  |  |  |
| Structural | 1804.955 | 1173 | 0.883 | 0.870 | 0.0791 | 0.0625 | — | — | — |
| Groups* | 1875.491 | 1218 | 0.878 | 0.870 | 0.0907 | 0.0625 | 70.536 | 45 | 0.009 |
| Pruned | 1844.537 | 1200 | 0.881 | 0.871 | 0.0846 | 0.0624 | 39.582 | 27 | 0.056 |
| Pruned & fixed | 1873.392 | 1220 | 0.879 | 0.871 | 0.0872 | 0.0623 | 68.437 | 47 | 0.091 |
| *Research Location* |  |  |  |  |  |  |  |  |  |
| Structural | 1467.571 | 868 | 0.889 | 0.875 | 0.0699 | 0.0603 | — | — | — |
| Groups | 1503.949 | 898 | 0.888 | 0.878 | 0.0723 | 0.0596 | 36.378 | 30 | 0.196 |
| Pruned | 1511.326 | 902 | 0.887 | 0.878 | 0.0732 | 0.0596 | 43.755 | 34 | 0.117 |
| *Research Type* |  |  |  |  |  |  |  |  |  |
| Structural | 1527.253 | 871 | 0.878 | 0.863 | 0.0699 | 0.0633 | — | — | — |
| Groups* | 1581.294 | 901 | 0.873 | 0.863 | 0.0756 | 0.0633 | 54.041 | 30 | 0.005 |
| Pruned | 1564.643 | 899 | 0.876 | 0.866 | 0.0740 | 0.0627 | 37.390 | 28 | 0.111 |
| Pruned & fixed | 1576.170 | 905 | 0.875 | 0.866 | 0.0756 | 0.0628 | 48.917 | 33 | 0.146 |

^*^Asterisks indicate statistically different groups (p < 0.05).

**Appendix S9. Latent intercepts for full and multigroup regression models**

Table S9. Latent intercepts for full and multigroup regression models.

|  | **Est.** | **Std.all** | **p** |
| --- | --- | --- | --- |
| *Full Model* |  |  |  |
| Attitude | 6.164 | 8.630 | <0.001 |
| Norm | 5.524 | 5.692 | <0.001 |
| Control | 3.329 | 4.488 | <0.001 |
| General intent | 2.246 | 1.392 | 0.002 |
| Sanitary processing | 4.809 | 4.187 | <0.001 |
| Good behaviours | 5.381 | 5.549 | <0.001 |
| PPE | 3.655 | 3.989 | <0.001 |
| Vaccines | 5.941 | 6.000 | <0.001 |
| *Career Stage* |  |  |  |
| *Student* |  |  |  |
| Attitude | 6.300 | 10.863 | <0.001 |
| Norm | 5.782 | 7.937 | <0.001 |
| Control | 3.247 | 4.849 | <0.001 |
| General intent | 5.750 | 3.637 | <0.001 |
| Sanitary processing | 6.132 | 4.931 | <0.001 |
| Good behaviours | 5.306 | 5.744 | <0.001 |
| PPE | 5.630 | 5.031 | <0.001 |
| Vaccines | 6.163 | 6.849 | <0.001 |
| *Early career* |  |  |  |
| Attitude | 6.292 | 9.778 | <0.001 |
| Norm | 5.444 | 5.350 | <0.001 |
| Control | 3.255 | 4.353 | <0.001 |
| Intent | 5.886 | 3.796 | <0.001 |
| Sanitary processing | 6.173 | 5.362 | <0.001 |
| Good behaviours | 5.467 | 4.528 | <0.001 |
| PPE | 5.696 | 6.159 | <0.001 |
| Vaccines | 6.077 | 6.207 | <0.001 |
| *Mid-career* |  |  |  |
| Attitude | 6.084 | 7.693 | <0.001 |
| Norm | 5.541 | 5.303 | <0.001 |
| Control | 3.376 | 5.089 | <0.001 |
| General intent | 5.705 | 3.568 | <0.001 |
| Sanitary processing | 6.110 | 5.542 | <0.001 |
| Good behaviours | 5.515 | 7.930 | <0.001 |
| PPE | 5.667 | 6.592 | <0.001 |
| Vaccines | 6.037 | 5.641 | <0.001 |
| *Late Career* |  |  |  |
| Attitude | 6.061 | 8.259 | <0.001 |
| Norm | 5.612 | 6.480 | <0.001 |
| Control | 3.332 | 4.631 | <0.001 |
| General intent | 5.844 | 3.956 | <0.001 |
| Sanitary processing | 6.251 | 5.749 | <0.001 |
| Good behaviours | 5.286 | 4.239 | <0.001 |
| PPE | 5.761 | 7.049 | <0.001 |
| Vaccines | 6.136 | 7.840 | <0.001 |
| *Research Type* |  |  |  |
| *Not disease-related* |  |  |  |
| Attitude | 6.154 | 8.841 | <0.001 |
| Norm | 5.470 | 5.787 | <0.001 |
| Control | 3.404 | 4.702 | <0.001 |
| General intent | 5.633 | 3.505 | <0.001 |
| Sanitary processing | 5.963 | 5.165 | <0.001 |
| Good behaviours | 5.199 | 5.033 | <0.001 |
| PPE | 5.509 | 5.964 | <0.001 |
| Vaccines | 6.040 | 5.725 | <0.001 |
| *Disease-related* |  |  |  |
| Attitude | 6.514 | 11.610 | <0.001 |
| Norm | 5.746 | 5.911 | <0.001 |
| Control | 3.048 | 4.647 | <0.001 |
| General intent | 6.507 | 7.584 | <0.001 |
| Sanitary processing | 6.551 | 7.922 | <0.001 |
| Good behaviours | 5.867 | 7.024 | <0.001 |
| PPE | 6.319 | 10.975 | <0.001 |
| Vaccines | 6.290 | 6.873 | <0.001 |
| *Both* |  |  |  |
| Attitude | 6.136 | 7.808 | <0.001 |
| Norm | 5.680 | 6.066 | <0.001 |
| Control | 3.231 | 4.734 | <0.001 |
| General intent | 5.765 | 3.298 | <0.001 |
| Sanitary processing | 6.496 | 6.335 | <0.001 |
| Good behaviours | 5.664 | 10.742 | <0.001 |
| PPE | 5.831 | 6.397 | <0.001 |
| Vaccines | 6.199 | 7.159 | <0.001 |

| **Appendix S10. Intercepts for variant measured indicators.**  Table S10. Intercepts for variant measured indicators. | | | |
| --- | --- | --- | --- |
|  | **Est.** | **Std.all** | ***P*** |
| *Research Location* |  |  |  |
| *Lower SES & Both* |  |  |  |
| Impracticality | -1.922 | -1.283 | <0.001 |
| Discomfort | -1.432 | -0.980 | <0.001 |
| Dedicated field clothes | 0.627 | 0.328 | 0.158 |
| *Higher SES* |  |  |  |
| Impracticality | 1.191 | 0.892 | 0.019 |
| Discomfort | 0.908 | 0.676 | 0.044 |
| Dedicated field clothes | 0.012 | 0.006 | 0.979 |

Though we also examined a third group—those who do fieldwork in both high- and low- socioeconomic status (SES) countries—there was no significant difference between this group and those who only work in low- SES countries. Therefore, we fixed these two groups to be equal and treated them as a single group (e.g., those who do any fieldwork in low-SES countries
